# Brain Reactions to Opening and Closing the Eyes: Salivary Cortisol and Functional Connectivity

**DOI:** 10.1007/s10548-022-00897-x

**Published:** 2022-06-06

**Authors:** Shen-Da Chang, Po-Chih Kuo, Karl Zilles, Tim Q. Duong, Simon B. Eickhoff, Andrew C. W. Huang, Arthur C. Tsai, Philip E. Cheng, Michelle Liou

**Affiliations:** 1 Institute of Statistical Science, Academia Sinica, Taipei, Taiwan; 2 Department of Computer Science, National Tsing Hua University, Hsinchu, Taiwan; 3 Institute of Neuroscience and Medicine (INM-1), Research Centre Jülich, Jülich, Germany; 4 Department of Radiology, Albert Einstein College of Medicine, New York, USA; 5 Institute of Systems Neuroscience, Medical Faculty, Heinrich Heine University Düsseldorf, Düsseldorf, Germany; 6 Institute of Neuroscience and Medicine (INM-7), Research Centre Jülich, Jülich, Germany; 7 Department of Psychology, Fo-Guang University, Yilan, Taiwan

**Keywords:** Approach-avoidance conflicts, Cortisol, DMN, Intraclass correlation, Ventral hippocampus

## Abstract

This study empirically assessed the strength and duration of short-term effects induced by brain reactions to closing/opening the eyes on a few well-known resting-state networks. We also examined the association between these reactions and subjects’ cortisol levels. A total of 55 young adults underwent 8-min resting-state fMRI (rs-fMRI) scans under 4-min eyes-closed and 4-min eyes-open conditions. Saliva samples were collected from 25 of the 55 subjects before and after the fMRI sessions and assayed for cortisol levels. Our empirical results indicate that when the subjects were relaxed with their eyes closed, the effect of opening the eyes on conventional resting-state networks (e.g., default-mode, frontal-parietal, and saliency networks) lasted for roughly 60-s, during which we observed a short-term increase in activity in rs-fMRI time courses. Moreover, brain reactions to opening the eyes had a pronounced effect on time courses in the temporo-parietal lobes and limbic structures, both of which presented a prolonged decrease in activity. After controlling for demographic factors, we observed a significantly positive correlation between pre-scan cortisol levels and connectivity in the limbic structures under both conditions. Under the eyes-closed condition, the temporo-parietal lobes presented significant connectivity to limbic structures and a significantly positive correlation with pre-scan cortisol levels. Future research on rs-fMRI could consider the eyes-closed condition when probing resting-state connectivity and its neuroendocrine correlates, such as cortisol levels. It also appears that abrupt instructions to open the eyes while the subject is resting quietly with eyes closed could be used to probe brain reactivity to aversive stimuli in the ventral hippocampus and other limbic structures.

**Supplementary Information:**

The online version contains supplementary material available at 10.1007/s10548-022-00897-x.

## Introduction

During resting-state functional magnetic resonance imaging (rs-fMRI), subjects remain relaxed with the eyes either closed (EC) or open (EO) without engaging in any task (Fox et al. 2009; Northoff et al. 2010). Several recent studies examining resting states have reported that operational conditions (i.e., eyes-closed, eyes-open, and eyes-open with fixation on an image) greatly affect the generalizability of scientific or clinical findings pertaining to connectivity networks, particularly in the sensory cortex (Andrews-Hanna et al. 2010; Feige et al. 2005; Fox et al. 2005; Kollndorfer et al. 2013; McAvoy et al. 2008; Patriat et al. 2013; Van Dijk et al. 2010; Yeo et al. 2011). In those studies, EC and EO conditions were employed in separate experimental runs, and the first 4 − 10 image volumes of the scanning session were discarded during the data preprocessing phase to allow for T1 equilibrium. Because the auditory instruction was normally brief enough, those discarded image volumes could have also minimized potential confounding effects from brain reactions to closing or opening the eyes. Nevertheless, abrupt auditory instructions could evoke functional states that deviate from previous accounts of resting states and have possible associations with motivation, anxiety and/or neuroendocrine activity (Henning et al. 2006; Yoshida et al. 2019). For example, an abrupt EO instruction could be aversive or novel and invade the consciousness of a subject when being relaxed quietly with eyes closed. Although research into EC/EO has uncovered interesting insights into rs-fMRI connectivity, there is a paucity of empirical data on temporal responses that occur immediately after closing/opening the eyes. There is also a lack of information pertaining to the roles played by neuroendocrine and psychological factors in the regulation of these responses. This study bridges this research gap by empirically assessing the strength and duration of short-term effects induced by brain reactions to closing/opening the eyes on a few well-known resting-state networks (RSNs) and the associations between these reactions and neuroendocrine activity.

Several RSNs have been identified corresponding to basic functions, such as vision, auditory perception, language, episodic memory, executive control and salience detection in the human brain (Damoiseaux et al. 2006; De Luca et al. 2006; Habas et al. 2009; Smith et al. 2009; Yeo et al. 2011). Six highly cited RSNs include the DMN (medial prefrontal cortex, posterior cingulate cortex, precuneus, lateral parietal cortex/angular gyrus, inferior temporal gyrus), fronto-parietal network (FPN; lateral prefrontal and inferior parietal cortices), salience network (SN; the anterior insula and anterior cingulate cortex), meso-paralimbic network (MPN; the amygdala, hippocampal formation, and temporal poles), visual network (VN; the primary and high level visual cortices), and sensorimotor network (SMN; the supplementary motor area, sensorimotor cortex, and secondary somatosensory cortex) (Syan et al. 2017). These networks have been identified primarily based on macroscopically defined regions of interest (ROIs). Some studies using the seed-based approach examined the basal ganglia, thalamus, and hypothalamus when investigating associations between cortisol levels and resting-state connectivity (Peters et al. 2019; Veer et al. 2012). Other studies examined the direct and indirect effects of neuroendocrine concentrations on different brain systems, such as the interaction between limbic structures (hippocampus and amygdala) and the prefrontal cortex (Albert and Newhouse 2019; Noack et al. 2019; Yoshida et al. 2019). The success of those studies has made it clear that any method used to investigate connectivity should be subjected to cross-validation by examining the interplay among connectivity networks, neuroendocrine systems, and psychological factors.

A number of recent studies have shown that movies or other naturalistic stimuli can synchronize brain responses among subjects, resulting in stable spatiotemporal activity patterns with a high degree of similarity (Hasson et al. 2010; Moraczewski et al. 2018; Nastase et al. 2019). In many of those studies, inter-subject correlation (ISC) analysis was used to assess the fMRI time courses that are consistently observed among subjects (Chen et al. 2016; Imhof et al. 2017). In ISC analysis, Pearson correlation is computed between two time-courses in a given ROI, the results of which are then applied to all pairs of subjects. The output values are Fisher Z-transformed, averaged, and finally re-transformed back to a correlation value indicating the average degree of similarity among subjects in terms of time courses pertaining to the given ROI (Schmälzle et al. 2017). Intraclass correlation (ICC), on the other hand, is applicable to cases involving more than a single pair of time-courses, and can be used to obtain a single ISC estimate without having to average multiple pairs of correlation values. Unlike Pearson correlation being scale-free, ICC is sensitive to mean- and variance-differences between time courses. Note that this is not necessarily a flaw, as these differences may reflect clinical severity or developmental changes. Theoretically, the asymptotic properties (e.g., standard error of estimation) of ICC are tractable. This makes the ICC approach a practical alternative to the averaging of correlation values across all pairs of subjects. Note also that ICC analysis is easily implemented within cytoarchitectonically (JuBrain) or macroscopically (AAL) defined ROIs, and remains computationally tractable even when dealing with a fairly large number of subjects.

As with fMRI experiments involving naturalistic stimuli, it is possible to synchronize brain responses among subjects simply by providing the same auditory instructions to keep EC-then-EO (or vice versa) for a specific duration. Individual ROIs in RSNs may exhibit high degrees of ISC when temporal responses induced by brain reactions to auditory instructions are incorporated in data analysis. Because a high degree of synchronicity is likely observed immediately after EC/EO onset and at no other times during resting-state scans, the time courses masked out by the ICC method may still convey information specific to individual subjects, varying as a function of psychological traits and cortisol levels. Within the context of well-known RSNs, our primary objective in the current study was to clarify the strength and duration of short-term effects induced by brain reactions to closing and opening eyes on the DMN, FPN, SN, and MPN, rather than to detail the issue of active versus activated processes under different resting states (Joel et al. 2011; Morcom and Fletcher 2007). We also considered EC/EO effects on the stress-response network (SRN; Lucassen et al. 2014) which is distributed in limbic structures as well as the orbitofrontal cortex. The SRN has been widely discussed in studies on the synthesis of rs-fMRI connectivity and neuroendocrine concentrations. We finally examined the neuroendocrine (cortisol) correlates in these networks to further validate our empirical findings.

## Methods

### fMRI Data Acquisition and Analysis

#### Participants and Tasks

A total of 55 right-handed young and healthy adults (27 males; average age 22.93 ± 3.08) were recruited for the rs-fMRI experiment and 36 of them had previous experiences performing tasks in an MRI scanner. The experiment was approved by the Human Participant Research Ethics Committee/Institutional Review Board at Academia Sinica (Taiwan) in accordance with the Declaration of Helsinki, and all subjects gave informed written consent before their enrollment into the study. During the fMRI experiment, each subject received exclusively auditory instructions to close or open the eyes through high-quality MR-compatible insert earphones (Sensimetrics Model S14) worn under hearing protection ear muffs. The 8-min experimental task in this study involved one 4-min cycle under the EC condition followed by one 4-min cycle under the EO condition (herein referred to as EC or EO onset). Under the EC condition, the subject was instructed to rest quietly with eyes closed, and the visual presentation was turned off. Under the EO condition, the subject was instructed to rest quietly with their eyes fixated on a central crosshair, and a red crosshair on a dark background image was presented using an MR-compatible visual stimulation system. Note that the order (EC-EO) was fixed based on the results of a pilot study involving 6 subjects.^1^ The pilot study revealed that this configuration was more relaxing for the subjects. We adopted the 4-min duration with the aim of minimizing motion artifacts, and stabilizing the strength of RSNs (Van Dijk et al. 2010).

#### Image Acquisition and Preprocessing

The fMRI scan was performed using a 3T MAGNETOM Skyra scanner (Siemens Healthcare, Erlangen, Germany) and a standard 20-channel head-neck coil. The echo planar imaging (EPI) scans were performed with parameters TR/TE = 2000-ms/30-ms, flip angle = 84°, 35 slices, slice thickness = 3.4 mm, FOV = 192 mm, and resolution 3 × 3 × 3.74 mm to cover the whole brain including the cerebellum. There were 120 image volumes collected per condition with a total of two conditions (240 volumes) in each session. In addition, four “dummy” volumes were acquired before the EC/EO paradigm to allow the MR signal to reach steady state, and those volumes were discarded by the scanner. T1-weighted anatomical images were also acquired with the following parameters: TR/TE = 2530-ms/3.30-ms, flip angle = 7°, 192 slices, FOV = 256 mm, and resolution 1 × 1 × 1 mm. The data were preprocessed using SPM12 (http://www.fil.ion.ucl.ac.uk/spm). In line with other rs-fMRI studies, empirical time courses were preprocessed by including motion correction, slice timing, linear detrending, and spatial transformation to the MNI-152 template (Ciric et al. 2017; Elliott et al. 2019; Satterthwaite et al. 2013). Nuisance regressors (e.g., motion parameters) were not included in the preprocessing phase to avoid inflated connectivity values (Caballero-Gaudes and Reynolds 2017; Nalci et al. 2019). Also, bandpass filtering (0.01–0.1 Hz) was not performed in the preprocessing phase for reasons detailed in the Discussion section.

Head motion was corrected by the SPM routine which estimated 6 motion parameters consisting of three translations and three rotations at each time point. The 6 parameters time series were represented by the framewise displacement (FD) and root-mean squared (RMS) displacement values relative to a single reference volume (Satterthwaite et al. 2013). The mean FD relative displacement (MRD) value was computed for each subject. The whole brain signal change was summarized by the derivative of RMS displacement over voxels (DVARS) (Power et al. 2011; Power et al. 2012). All the displacement values were computed by the BRAMILA tools available at (https://users.aalto.fi/~eglerean/bramila.html). There were three subjects whose parameters exceeded 2-mm and ${2}^{\circ }$. Their image volumes were manually divided into two segments. Realignment was performed in accordance with a new reference volume formulated for each segment. The aligned image volumes within individual segments were co-registered individually to the standard MNI template. Table 1 provides a summary of mean and standard deviation of MRD and DVAR values of the 55 subjects along with the subgroup (ten subjects) with the highest motion parameters and the subgroup (ten subjects) with the lowest motion parameters.

**Table 1:** Motion parameters in the complete group and subgroups

Samples	Sizes	MRD, mm (SD)	DVAR, % (SD)
Complete group	55 (27 males)	0.143 (0.057)	0.419 (0.052)
Low-motion subgroup	10 (5 males)	0.086 (0.007)	0.402 (0.030)
High-motion subgroup	10 (5 males)	0.229 (0.068)	0.435 (0.071)

The T1-weighted high-resolution image volume was co-registered to the mean of the realigned EPI images and was spatially normalized with voxel size 2 × 2 × 3 mm to the standard MNI-152 space (Note: The anisotropic voxel size was chosen to reflect the resolution of raw data). The signal-to-noise ratio of functional images was enhanced by spatial smoothing using a 4-mm FWHM (full width at half maximum) Gaussian kernel.

#### Data Analysis

##### ICC Index

In fMRI applications, two ICC indices have been widely used to assess test–retest reliability between brain activation maps and connectivity networks: ICC(C, M) and ICC(A, M), where M denotes the number of experimental replicates, C refers to consistency, and A refers to agreement (Brandt et al. 2013; Caceres et al. 2009; Fiecas et al. 2013; Kristo et al. 2014; Termenon et al. 2016; Wang et al. 2017; Zanto et al. 2014; Zhu et al. 2014). ICC(C, M) is insensitive to mean differences in intensity among subjects, and therefore gives a slightly higher ISC value when MR images are acquired using different scanners. The empirical values of the indices range from − ∞ to 1 (Lahey et al. 1983). The magnitude of ICC indices is determined by many factors, including the size of M. When computed using M of different sizes, the indices cannot be directly compared with one another unless they are normalized according to standard errors. The standard error of ICC(C, M) for temporally dependent time courses was derived under a stationarity assumption (Kuo et al. 2019). In the sequel, the asymptotic standard error of ICC(A, M) is derived by an analogous approach. In a single voxel, the data forms an n(row)-by-M(column) matrix of image intensities, where the M columns indicate subject-level time courses with n time points in each column. Let **S** denote an M-by-M column-wise cross-covariance matrix with zero lagged time points among the preprocessed fMRI time courses, and let **V** denote an n-by-n row-wise covariance matrix. An off-diagonal element in **S** is computed using a pair of time courses from two subjects as a reflection of their synchronization strength (or similarity). An off-diagonal element in **V** is computed using a pair of intensity vectors between two points in time as an indication of whether between-subject changes in intensity remain the same at the two time points. The ICC(A, M) or agreement index introduces a mean-sensitive correlation measure by considering the variability in mean intensity values among M subjects and among n time points (McGraw and Wong 1996).

The index has been widely applied in clinical research when investigating test–retest reliability of imaging techniques and connectivity networks (Fiecas et al. 2013; Kristo et al. 2014; Zanto et al. 2014). The index can be expressed as follows:

1 $$\widehat{\text{A}}={ (\frac{\text{M}}{\text{M}-1})[ {\underline{1}}^{{^{\prime}}}}\mathbf{S}\underline{1}-\text{tr}\left(\mathbf{S}\right) ]/\left\{{\underline{1}}^{{^{\prime}}}\mathbf{S}\underline{1} \left[ 1+\left(\frac{\left(\text{n}-1\right){\varvec{\Gamma}}}{\text{n}\left(\text{M}-1\right)}\right)-\left(\frac{\text{M}}{\text{n}\left(\text{M}-1\right)}\right)\frac{\text{tr}\left(\mathbf{S}\right)}{{\underline{1}}^{{^{\prime}}}\mathbf{S}\underline{1}}+ \frac{1}{\text{n}\left(\text{M}-1\right)}\right]\right\},$$

which is also identical to the ICC(2, M) index in Shrout and Fleiss (1979). A large and positive $\widehat{\text{A}}$ value indicates that the subject-to-subject variation in their time courses is small; the size of $\widehat{\text{A}}$ is decreased, on the other hand, if MR images are acquired from different scanners or brain reactivity is unique to individual subjects. In the denominator of (1), **Γ** denotes the ratio between $\left({\underline{1}}^{{^{\prime}}}\mathbf{V}\underline{1}/{\text{n}}^{2}\right)$ and $\left({\underline{1}}^{{^{\prime}}}\mathbf{S}\underline{1}/{\text{M}}^{2}\right)$ where $\underline{1}$ is the summing vector of order M with the transpose ${\underline{1}}^{^{\prime}}$, and tr(**S**) denotes the trace of **S**.

Based on the law of large numbers, the **Γ** ratio consistently estimates the ratio γ = ${\sigma }_{r}^{2}$/${\upsigma }_{\text{c}}^{2}$ as n and M become large. The parameters ${\sigma }_{r}^{2}$ and ${\sigma }_{c}^{2}$ are the mean values of elements in **V** and **S**, respectively. The $\widehat{\text{A}}$ index can be approximated by

2 $$\widehat{\text{A}}\simeq { \left(\frac{\text{M}}{\text{M}-1}\right)[ {\underline{1}}^{^{\prime}}}{\varvec{S}}\underline{1}-\text{tr}\left({\varvec{S}}\right) ]/\left\{{\underline{1}}^{^{\prime}}{\varvec{S}}\underline{1} \left[1+\left(\frac{1}{\text{M}-1}\right){\varvec{\Gamma}}+O\left(\frac{1}{\text{nM}}\right)\right]\right\} \simeq \widehat{\text{C}}\left[\frac{\text{M}-1}{\left(\text{M}-1\right)+{\varvec{\Gamma}}}\right]+\text{O}\left(\frac{1}{\text{nM}}\right)$$

In (2), O $\left(\frac{1}{\text{nM}}\right)$ denotes a remainder term converging to zero with order n times M (Note: The proof can be found in Table A in the Supplementary Materials). The $\widehat{\text{C}}$ index in (2) denotes the estimate of ICC(C, M) index, which is identical to ICC(3, M) proposed by Shrout and Fleiss (1979). Because ${\varvec{\Gamma}}$ is a positive value, the range of $\widehat{\text{A}}$ is smaller than that of $\widehat{\text{C}}$; the ${\varvec{\Gamma}}$ value is affected by the mean differences in intensity among subjects. The variance of $\widehat{\text{C}}$ has been given in the literature (Browne 1974; Kuo et al. 2019; van Zyl et al. 2000); the asymptotic result in (2) suggests that the variance of $\widehat{\text{A}}$ can be approximated by

3 $${\text{Var}}(\widehat{\text{A}}) \backsimeq \text{Var}(\widehat{\text{C}}){\left(\frac{\text{M}-1}{\left(\text{M}-1\right)+\upgamma }\right)}^{2}{|}_{\gamma =\Gamma }$$

In applications, γ can be evaluated at Γ. The matlab program for computing Var($\widehat{\text{C}}$) is available at (https://github.com/PoChihKuo/icc-neuroimage), which has the following form:

4 $${\text{Var}}\left( {\widehat{{\text{C}}}} \right) = 2{\text{n}}^{{ - 1}} \left. {\eta ^{\prime}{\text{K}}_{M} \left( {\Sigma \otimes \Sigma } \right){{\text{K}^{\prime}}_{M}} {\eta} } \right|_{{\sum { = S} }} ,$$

where ${\eta }^{^{\prime}}$ is the derivative of ICC(C, M) with respect to ${\text{vech}}^{\prime}{\varvec{\Sigma}}$ = $({\text{vech}}{\varvec{\Sigma}})^{\prime},$ and $\otimes$ is the Kronecker product. ${\varvec{\Sigma}}$ is the population counterpart of ***S***; ${\text{vech}}{\varvec{\Sigma}}$ is the half-vectorization of the matrix **Σ**; ${\text{K}}_{M}$ is the transformation matrix of order M(M + 1)/2-by-M^2^, with the identity ${\text{vech}}{\varvec{\Sigma}}={\text{K}}_{M}({\text{vec}}{\varvec{\Sigma}})$, where ${\text{vec}}{\varvec{\Sigma}}$ is the vectorization of the matrix **Σ** and is of order M^2^-by-1 and ${\varvec{\Sigma}}$ can be evaluated at ***S*** in application. The derivative can be expressed as

5 $$\eta ^{\prime} = \frac{{\text{M}}}{{({\text{M}} - 1)({\mathbf{\underset{\raise0.3em\hbox{$\smash{\scriptscriptstyle-}$}}{1}^{\prime} }}\Sigma {\mathbf{\underset{\raise0.3em\hbox{$\smash{\scriptscriptstyle-}$}}{1} }})^{2} }}\left. {\left[ { - ({\mathbf{\underset{\raise0.3em\hbox{$\smash{\scriptscriptstyle-}$}}{1}^{\prime} \Sigma \underset{\raise0.3em\hbox{$\smash{\scriptscriptstyle-}$}}{1} }}){\text{vec}^{\prime}}({\text{I}}_{M} ) + {\text{tr}}({\mathbf{\Sigma }})({\mathbf{\underset{\raise0.3em\hbox{$\smash{\scriptscriptstyle-}$}}{1}^{\prime} }} \otimes {\mathbf{\underset{\raise0.3em\hbox{$\smash{\scriptscriptstyle-}$}}{1}^{\prime} }})} \right]{\text{G}}_{M} } \right|_{{\sum { = S} }} ,$$

where I_*M*_ is the identity matrix of order M, and ${\text{G}}_{M}$ is the transformation matrix of order M^2^-by-M(M + 1)/2 with the identity ${\text{vec}}{\varvec{\Sigma}}={\text{G}}_{M}\text{(vech}{\varvec{\Sigma}}$). The *t*-value or standardized value for evaluating the empirical agreement index for significance is defined as

6 $${t}_{\widehat{\text{A}}}=\frac{\widehat{\text{A}}}{\sqrt{\text{Var}\left(\widehat{\text{A}}\right)}}$$

The data acquisition provided n = 240 and M = 49 in ISC analysis. A schematic illustration of computing the agreement index, error variance, and ${t}_{\widehat{\text{A}}}$ value in the ICC method can be found in Fig. A-1 in the Supplementary Materials.

##### Statistical Evaluation of the Stationarity Assumption

The derivation of $\widehat{A}$ in (2) follows from the law of large numbers (i.e., the sample size n or M is reasonably large). The distribution approximations in (3) and (4) are valid when voxel responses $\left\{{X}_{k,i}: 1 \le k \le n, 1 \le i \le M\right\}$ across the n image scans have bounded fourth moments and satisfy two basic conditions: (i) strictly stationary along the sequence of image scans, and (ii) ρ-mixing which satisfies the condition that the maximal modulus of lagged correlation values between ${X}_{k,i}$ and ${X}_{k+\upsilon , i}$ converge toward 0, that is, for 1 ≤ *k* ≤ n–υ and 1 ≤ *i* ≤ M, or max _*k*, *i*_$\left|\rho \left( {X}_{k,i},{X}_{k+\upsilon , i}\right)\right|$ → 0 as υ → ∞. Fig. A-2 in the Supplementary Materials provides a graphical proof for the ρ-mixing condition, which shows the bivariate plots of the maximal size of lagged-correlation coefficients against the increasing sequence 10 ≤ υ ≤ 230 given the maximal length n = 240. In the plots, it is clear that the lagged sample correlation coefficients decrease from 0.30 to 0.13 as υ is increasing, suggesting that it can decrease toward 0 when υ increases toward infinity.

It follows from Theorem 1.3 in Bradley (2012) that the stationary elements ${s}_{i,j}$ satisfy asymptotic normality of ***S***, which is the basis for deriving the asymptotic variance of $\widehat{\text{C}}$ in (4). Several empirical studies have suggested that rs-fMRI time courses tend to be non-stationary (Cole et al. 2010; Lee et al. 2013), which may restrict the use of Var($\widehat{\text{A}})$ in (3). When temporal responses induced by brain reactivity to closing and opening the eyes are incorporated into data analysis, however, the problem of non-stationarity might become less relevant. To validate the application of (3) and (4), stationarity condition (i) must be examined. The Kwiatkowski–Phillips–Schmidt–Shin (KPSS) test was applied to individual time courses in all in-brain voxels for evaluating the null hypothesis that time courses are trend-stationary against the alternative of a unit root (Kwiatkowski et al. 1992). In addition, the Augmented Dickey–Fuller (ADF) test was also applied to evaluate the null hypothesis of non-stationarity against the alternative hypothesis of stationarity. A time course can be assumed to be stationary if the KPSS test fails to reject the null hypothesis, and the ADF test rejects the null hypothesis. Among in-brain voxels in the current study, a small portion of time courses (2.02%) were consistently rejected by the KPSS test in at least 70% subjects (4.82% in 60% and 11.79% in 50% subjects). After the weak FDR control, the proportion was reduced to 1.96% in at least 70% subjects (4.64% in 60% and 11.14% in 50% subjects). The rejected time courses were mainly distributed in the subgenual anterior cingulate (Areas s24 and s32), orbitofrontal cortex (Areas Fo1, Fo2, and Fo3), and frontal pole (Fp1, Fp2). None of the time courses was consistently accepted by the ADF test across subjects (i.e., 0.15% time courses was consistently accepted in five subjects). Based on the results, the stationarity condition (i) was found valid in most brain voxels.

##### ICC Maps

As demonstrated by the stationarity tests, the time courses of in-brain voxels generally satisfied the assumption of stationarity, which partially validated the use of Fourier phase randomization to generate surrogate data for thresholding the empirical ${t}_{\widehat{\text{A}}}$ values when determining statistical significance of these values (Lerner et al. 2011). Familywise Type-I error rate was controlled at α = 0.05 using the false discovery rate (FDR) control procedure (Benjamini and Hochberg 1995; Benjamini and Yekutieli 2001; Genovese et al. 2002; Langers et al. 2007). In the FDR procedure, a sequence of ordered *p*-values (i.e., the probability of randomly simulated *t* values in the surrogate distributions greater than or equal to the observed ${t}_{\widehat{\text{A}}}$ values) were compared with the critical value (*i*/K)[α/C(K)] for the *i-*th *p*-value in the ordered sequence to control the FDR at α, where K is the total number of voxels considered and C(K) is a predetermined constant. The choice of constant depends on the joint distribution of *p*-values in the sequence. It has previously been established that FDR control may increase the false-positive or false-negative rate depending on the overall proportion of voxels that are truly synchronized among subjects (Chumbley et al. 2010). In the current study, C(K) = ${\sum }_{i=1}^{K}{i}^{-1}$ was selected for a weak control of the false positive rate (Genovese et al. 2002). The ICC maps were constructed by identifying the supra-threshold voxels with the ${t}_{\widehat{\text{A}}}$ values exceeding the FDR critical values. The supra-threshold maps were co-registered to the JuBrain Cytoarchitectonic Atlas available at (http://www.fz-juelich.de/inm/inm-7/tools) for the topographical definition of area-specific effects (Eickhoff et al. 2005).

##### Types of Short-Term Effects

Table 2 presents a list of cytoarchitectonic areas involved in the DMN, MPN, FPN and SN and their corresponding Brodmann nomenclature. We annotated brain areas linked to the DMN in a meta-analysis of nine Positron Emission Tomography (PET) studies (132 subjects) by Shulman et al. (1997). After re-analyzing the PET data, Buckner et al. (2005) identified DMN areas that were more active during passive tasks than during active tasks (See Figure 2 in Buckner et al. (2008)). Zilles (2017) included anatomical correspondence between the DMN in Buckner et al. (2008) and cytoarchitectonic areas in JuBrain. In the current study, we also considered cortisol levels, and therefore examined brain structures associated with the SRN (Cerqueira et al. 2008; Lucassen et al. 2014), including the orbitofrontal cortex, hippocampus, entorhinal cortex, amygdala, nucleus accumbens, basal forebrain, and subgenual anterior cingulate cortex. Brain areas unavailable in JuBrain were defined using the Neuromorphometrics Inc. Atlas (http://neuromorphometrics.com) and MRIcro software (http://www.mricro.com) in support of network analysis.

**Table 2:** Cytoarchitectonic brain areas referred to in the connectivity networks

Abbreviations	Brain areas	Relationship with brodmann areas	Atlases (ICNs)	References
HC	Cornu ammonis (CA1-3), dentate gyrus (DG) including Fascia dentate (FD) and CA4, Subicular complex (SC), and hippocampal-amygdaloid transition area (HATA) in the hippocampus	n.d	JuBrain	Amunts et al. (2005); Palomero-Gallagher et al. (2020); areas in HC are part of DMN (Shulman et al. 1997), MPN (Syan et al. 2017), and SRN (Lucassen et al. 2014)
AM	Centro-medial (CM), superficial (SF), inferior (IF) and medial fiber bundles (MF), laterobasal (LB) and ventro-medial nuclei (VTM) of the amygdala, and amygdalostriatal transition area (Astr)	n.d	JuBrain	Amunts et al. (2005); AM nuclei are part of MPN and SRN (Lucassen et al. 2014; Syan et al. 2017)
NA	Nucleus accumbens	n.d	Neuromorphometrics, Inc	http://neuromorphometrics.com; NA is part of SRN (Lucassen et al. 2014)
AIns	Anterior Insula	13	Neuromorphometrics, Inc	http://neuromorphometrics.com; BA13 is part of SN (Syan et al. 2017)
BF	Nucleus Ch1(medial septal nucleus), Ch2, Ch3, and Ch4 (basal nucleus of Meynert) in the basal forebrain	n.d	JuBrain	Zaborszky et al. (2008); Nucleus in BF are part of SRN (Lucassen et al. 2014)
EnC	Entorhinal cortex	28	JuBrain	Amunts et al. (2005); EnC is part of DMN (Shulman et al. 1997), MPN (Syan et al. 2017), and SRN (Lucassen et al. 2014)
EcC	Ectorhinal cortex (Parahippocampal cortex)	36	Neuromorphometrics, Inc	http://neuromorphometrics.com; EcC is part of DMN (Shulman et al. 1997), and MPN (Syan et al. 2017)
SPC	Areas 5L, 5M, 5Ci, 7PC, 7A, 7P, 7M in the superior parietal cortex	5, 7	JuBrain	Scheperjans et al. (2008); BA5 and BA7 are part of DMN (Syan et al. 2017)
FEF	Frontal eye field	Part of 8 at the junction of the superior frontal and precentral sulci	MRIcro	http://www.mricro.com. BA8 is part of FPN (Hutchison et al. 2012)
DLPC	Dorsolateral prefrontal cortex	9, 46	MRIcro	http://www.mricro.com; BA9 is part of DMN (Shulman et al. 1997); BA9 and BA46 are part of FPN (Syan et al. 2017)
FP	Areas Fp1 and Fp2 in the frontal pole	10	JuBrain	Bludau et al. (2014); Fp1 and Fp2 are part of DMN (Shulman et al. 1997; Syan et al. 2017)
OFC	Areas Fo1, Fo2, and Fo3 in the orbito-frontal cortex	11	JuBrain	Henssen et al. (2016); Fo1, Fo2, and Fo3 are part of DMN (Shulman et al. 1997; Syan et al. 2017) and SRN (Lucassen et al. 2014)
sACC	Subgenual anterior cingulate cortex	Subgenual parts of 24 and 32 plus 25 and 33	JuBrain	Palomero-Gallagher et al. (2009); s24 and s32 are part of DMN (Shulman et al. 1997); s24, the infralimbic (Area 25), and prelimbic (Area s32) cortices are part of SRN (Lucassen et al. 2014); ACC is part of SN (Syan et al. 2017)
pACC	Pregenual anterior cingulate cortex	Pregenual parts of 24 and 32	JuBrain	Palomero-Gallagher et al. (2009); p24 and p32 are part of DMN (Shulman et al. 1997); ACC is part of SN (Syan et al. 2017)
TP	Temporal pole	38	Neuromorphometrics, Inc	http://neuromorphometrics.com; TP is part of DMN (Shulman et al. 1997), and MPN (Syan et al. 2017)
ITG	Inferior temporal gyrus	20	Neuromorphometrics, Inc	http://neuromorphometrics.com; ITG is part of DMN (Shulman et al. 1997; Syan et al. 2017)
MTG	Middle temporal gyrus	21	Neuromorphometrics, Inc	http://neuromorphometrics.com; MTG is part of DMN (Shulman et al. 1997)
vPCC	Ventral posterior cingulate	23	MRIcro	http://www.mricro.com; vPCC is part of DMN (Shulman et al. 1997; Syan et al. 2017)
PCu	Dorsal posterior cingulate (Precuneus)	31	MRIcro	http://www.mricro.com; PCu is part of DMN (Shulman et al. 1997; Syan et al. 2017)
AG	Areas PGa and PGp in the angular gyrus	39	JuBrain	Caspers et al. (2008); PGa and PGp are part of DMN (Shulman et al. 1997; Syan et al. 2017), and FPN (Syan et al. 2017)
SG	Areas PF, PFm, PFcm, PFop, and PFt in the supramarginal gyrus	40	JuBrain	Caspers et al., (2008); Areas PFm and PFt are part of DMN (Shulman et al. 1997); BA40 is part of FPN (Syan et al. 2017)
BR	Broca’s region	44, 45	JuBrain	Amunts et al. (1999). BA44 and BA45 are part of FPN (Syan et al. 2017)
OrIFG	Orbital part of the inferior frontal gyrus	47	Neuromorphometrics, Inc	http://neuromorphometrics.com; BA47 is part of FPN (Syan et al. 2017)

*n.d.* denotes not determined

For each ROI (e.g., Area 25 in the sACC) in Table 2, we identified supra-threshold voxels with significant ${t}_{\widehat{A}}$ values. We initially classified the corresponding mean time courses across subjects within each target ROI using iterative K-means clustering and the Silhouette statistics. This was meant to ensure that time courses were homogeneous within each ROI. In cases where an ROI contained two types of time courses (e.g., anterior insula), the ROI was further partitioned into two sub-ROIs. Following an initial examination, the time courses were averaged within each ROI or sub-ROI at the subject level for use in deriving connectivity values. Mean time courses at the subject-level were pooled and averaged at the group level (i.e., one mean time course in each ROI or sub-ROI) for classification into homogeneous networks using iterative K-means clustering (i.e., This procedure was to identify various types of short-term effects). The optimal size for K (number of networks) was determined using the Silhouette statistic. Interested readers may refer to p.88 in Kaufman and Rousseeuw (2009) for a qualitative interpretation of the Silhouette statistic.

Correlation values were computed using subject-level mean time courses between each pair of ROIs within a given network or cluster (Betti et al. 2013; O'Neil et al. 2014; Wang et al. 2007) and between two networks. Fisher's Z transformation was used to enhance the Gaussianity of correlation values. We identified 55 Fisher Z-transformed correlation values between each pair of ROIs (each connectivity value), the sample mean of which was evaluated for statistical significance using the Student’s t-test (Betti et al. 2013; O'Neil et al. 2014; Wang et al. 2007) with the weak FDR control of family-wise error rates. To facilitate visualization, connectivity networks were reconstructed using the BrainNet Viewer (Xia et al. 2013). Although K-means clustering was used only to identify distinct networks associated with group-level mean time courses, we also plotted subject-level mean time courses to enable the visualization of individual differences.

### Neuroendocrine Data: Acquisition and Analysis

#### Participants

Due to budget considerations, saliva samples were obtained before/after the fMRI scans from 25 (12 males; average age 23.44 ± 2.52) of the 55 subjects with the aim of validating the connectivity networks derived from ISC analysis based on their associations with neuroendocrine activity. After one cycle of EC-EO (or EO-EC given to the 6 pilot subjects) resting-state scanning, subjects were exposed to 20-min natural sound and speech stimulation. Note however that only resting-state time courses were considered in the current study. Note also that the pre-scan saliva samples were collected at least 10-min before the fMRI scan; therefore, the interval between the collection of pre-scan and post-scan saliva samples was at least 40-min (Pruessner et al. 2008).

#### Demographic Factors

The literature on rs-fMRI includes numerous references describing the effects of stress and anxiety on cortical and limbic structures (Hanson et al. 2019; Morgenroth et al. 2020; Olson et al. 2019; Torrisi et al. 2019). Subjects in the saliva group provided personal information (gender and age) and completed the 40-item State-Trait Anxiety Inventory (STAI) (Spielberger 2010), and the 25-item Stress Questionnaire of the ISMA (SQ-ISMA) available at (http://isma.org.uk/wp-content/uploads/2013/08/Stress-Questionnaire.pdf) (Byrne et al. 2007; Levenstein et al. 1993) prior to the fMRI scan session. The STAI has been used to assess explicit anxiety levels (Shek 1993; Tyc et al. 1995) as a reflection of the degree of mental tension induced by stressful experiences, predicted future dangers, or anticipated failures. The 20-item STAI-Trait scale measures stable attributes indicating how a subject generally feels, whereas the 20-item STAI-State scale assesses how a subject feels at the time s/he takes the inventory. The total number of ‘yes’ answers to items in the SQ-ISMA indicates the incidence of stress symptoms. The total score can be compared with a reference table attached to the questionnaire; for example, a score of ≥ 14 implies that a subject is highly prone to stress (i.e., s/he demonstrates many traits that can lead to unhealthy behavior).

#### Salivary Hormone Assays

Saliva samples were obtained between 9 and 11 AM to prevent circadian changes in hormone concentrations. The saliva samples were obtained by having the subject chew a cotton ball for at least 1-min. The collected cotton specimens were stored in microcentrifuge tubes to avoid contamination. Saliva extraction involved centrifuging the tubes at 3000 × *g* for 15-min. After the supernatant was withdrawn, the samples were centrifuged at 3000 × *g* for 15-min one more time, and then stored at ${-20}^{^\circ }$ C until assayed. Cortisol levels were measured in duplicate aliquots using commercial immunoassay kits with 96-well microtiter plates raised against cortisol (Item No. 500360, Cayman Chemical, USA). Hormone levels were measured using a photometric microplate reader (Metertech company, Taiwan) at 405 nm. The sensitivity of the kits was 40 pg/ml, which was below the lowest detectible value in the whole experiment (Note: Hormone levels in saliva samples are expressed as pg/ml). The coefficients of variation between duplicates within and between assays were below 6.0%. Cortisol levels were measured in the pre-scan and post-scan saliva samples (herein denoted as pre-cortisol and post-cortisol, respectively).

#### Statistical Analysis

There were 25 Fisher Z-transformed correlation values between each pair of ROIs in Table 2 (e.g., between Areas 25 and 33). In each subject, the Z scores were averaged for the analysis of left-, right-, and inter-hemispheric connectivity within each network and between two networks. Partial correlation values were computed between cortisol levels and connectivity values by controlling for demographic factors.

## Results

**Fig. 1:**
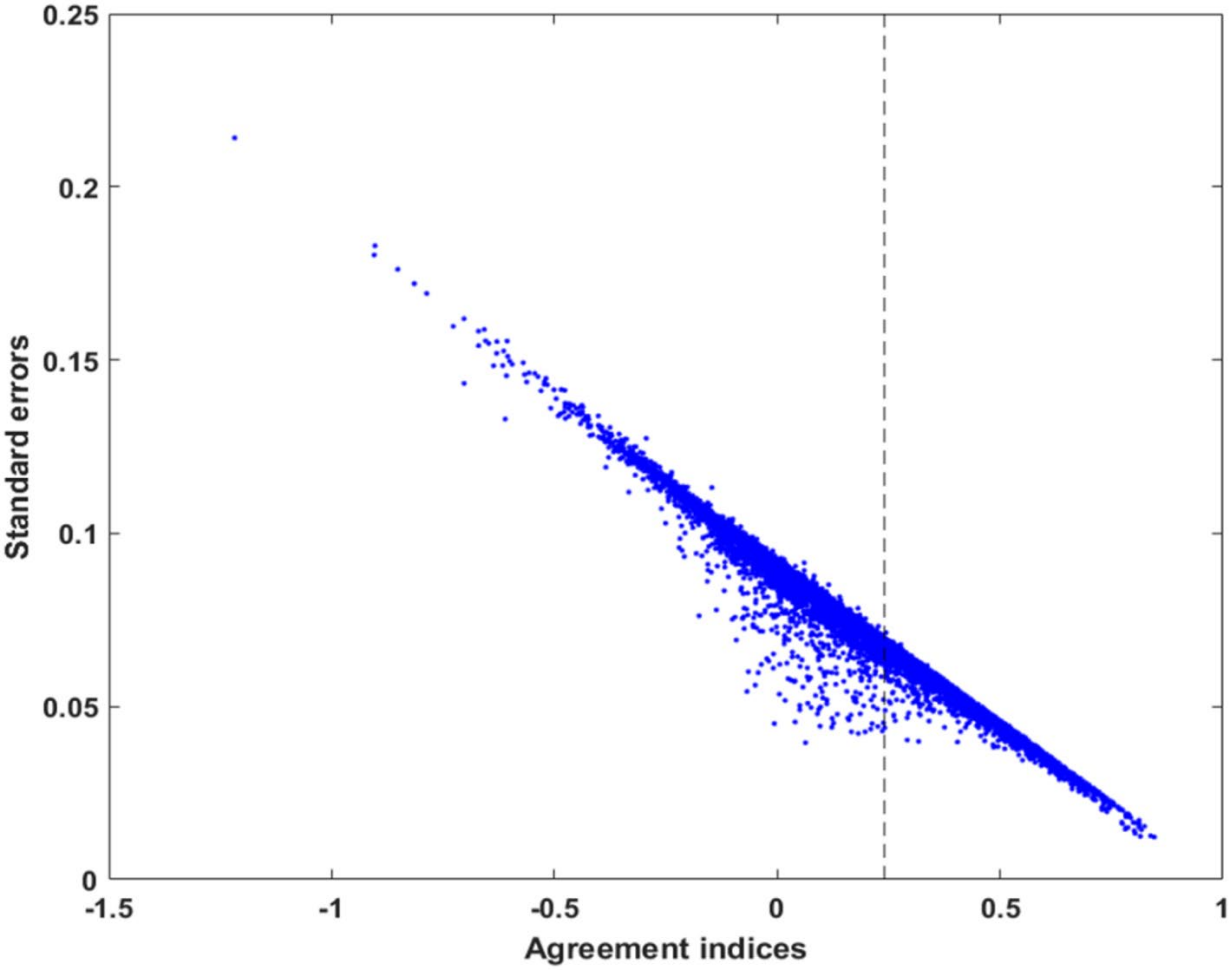
The bivariate plots of agreement indices versus their standard error estimates. Supra-threshold voxels after FDR control had $\widehat{A}$ ≥ 0.243 which lie on the right-hand side of the dashed line

**Fig. 2:**
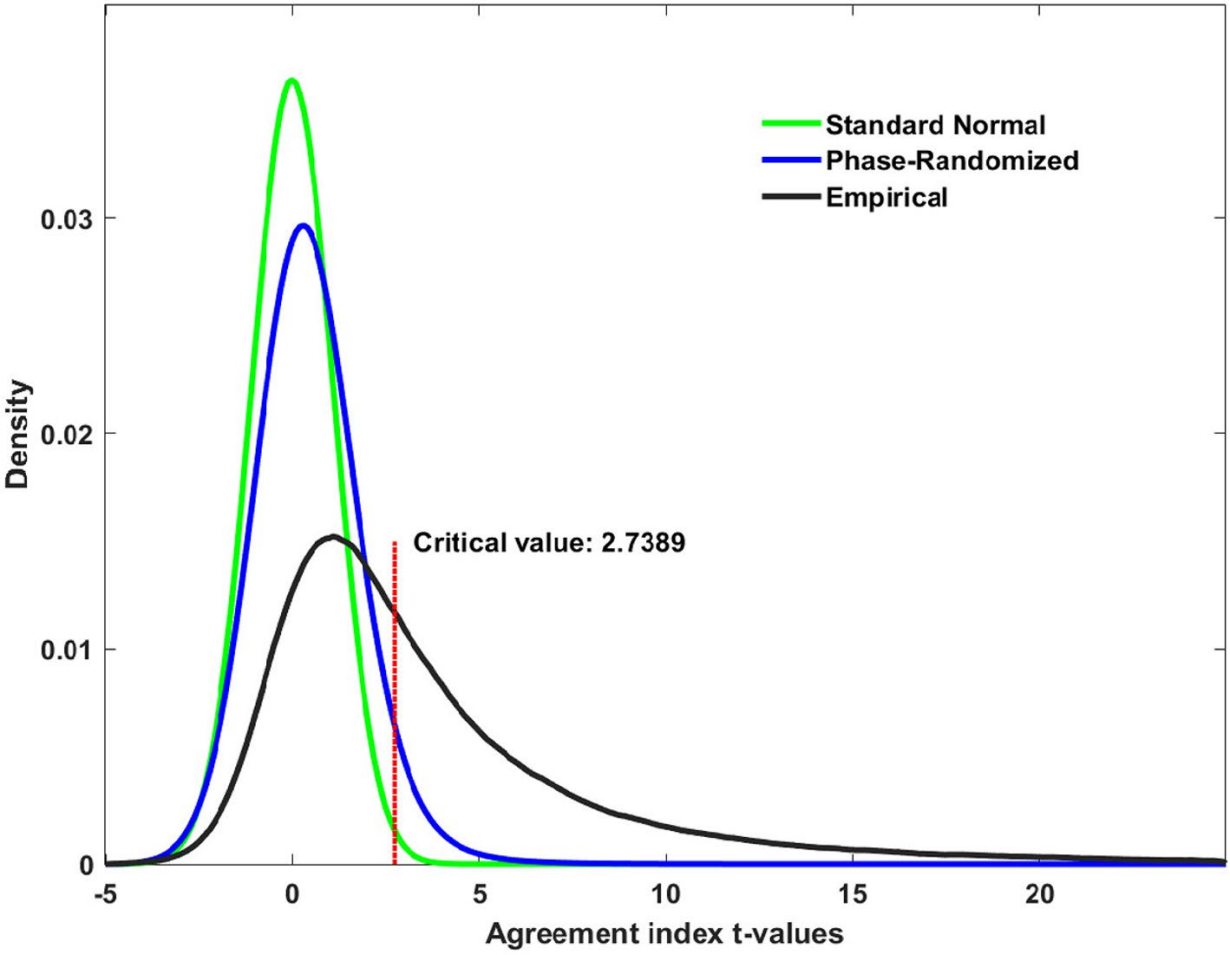
The distributions of phase-randomized and empirical ${t}_{\widehat{\text{A}}}$ values for in-brain voxels. The tail probability 0.05 has the corresponding critical value 2.7389 in the phase-randomized distribution

Figure 1 presents bivariate plots of $\widehat{A}$ values arranged according to standard errors. After FDR control, the supra-threshold voxels presented $\widehat{A}$ ≥ 0.243; that is, falling on the right side of the dashed line in the figure. These plots suggest that $\widehat{A}$ values are inversely proportional to the standard error estimates. After testing stationarity and ρ-mixing assumptions, it is possible to compare the ideal 1/$\sqrt{n}$ convergence rate of the standard error estimates in Eq. (4) vs. empirical values. Here, the average standard error estimates in the plot is 0.0727 given the sample size n = 240 and range of the standard error estimates (0.01, 0.2). These empirical estimates support the use of Eq. (4) for the standardization of $\widehat{A}$ values (i.e., ${t}_{\widehat{\text{A}}}$), based on stationary and weakly dependent fMRI time courses (Bradley 2012). Figure 2 displays the phase randomization distribution which was constructed using 50 surrogate samples based on in-brain voxels (Note: More than 50 surrogate samples did not significantly improve the ICC maps). The distribution of empirical ${t}_{\widehat{\text{A}}}$ values of in-brain voxels are also given in the same figure. As a reference, the supra-threshold ICC maps based on significant ${t}_{\widehat{\text{A}}}$ values can be found in Fig. B in the Supplementary Materials.

### EC/EO Effects

Based on the group-level mean time courses in ROIs listed in Table 2, our use of K-means clustering and the Silhouette statistic identified three networks (or three types of short-term effects), which showed distinct patterns following auditory EC/EO instructions. Among the three, one network showed increased (i.e., positive) activity after the onset of auditory instructions, whereas ROIs in this network presented considerable overlap with brain areas in the DMN, FPN, and SN. Herein, it is referred to as the transition-positive network (TPN) which engaged brain areas in the (i) dorsolateral prefrontal cortex (DLPC), (ii) posterior cingulate cortex (vPCC and PCu), (iii) left frontal eye field (FEF; 20% versus 4% supra-threshold voxels in the left and right hemispheres), (iv) pregenual anterior cingulate cortex (pACC; p24ab, p24c, and p32), (v) superior parietal cortex (5M, 7P, and 7M in bilateral hemispheres, along with 5Ci in the left hemisphere; 74% supra-threshold voxels in 5M were classified within this network), (vi) rostral angular gyrus (PGa), (vii) supramarginal gyrus (SG; PF and PFm in bilateral hemispheres, along with PFcm, PFop, and PFt in the left hemisphere), (viii) anterior insula (AIns; 72% supra-threshold voxels in AIns were classified within this network), (ix) left orbital part of the inferior frontal gyrus (OrIFG; 19% versus 3% supra-threshold voxels in the left and right hemispheres), and (x) Broca’s region (BR; 44 and 45). Most of ROIs within this network exhibited more supra-threshold voxels in the left hemisphere compared with the right hemisphere, for example, Areas 7P, PF, and 44.

**Fig. 3:**
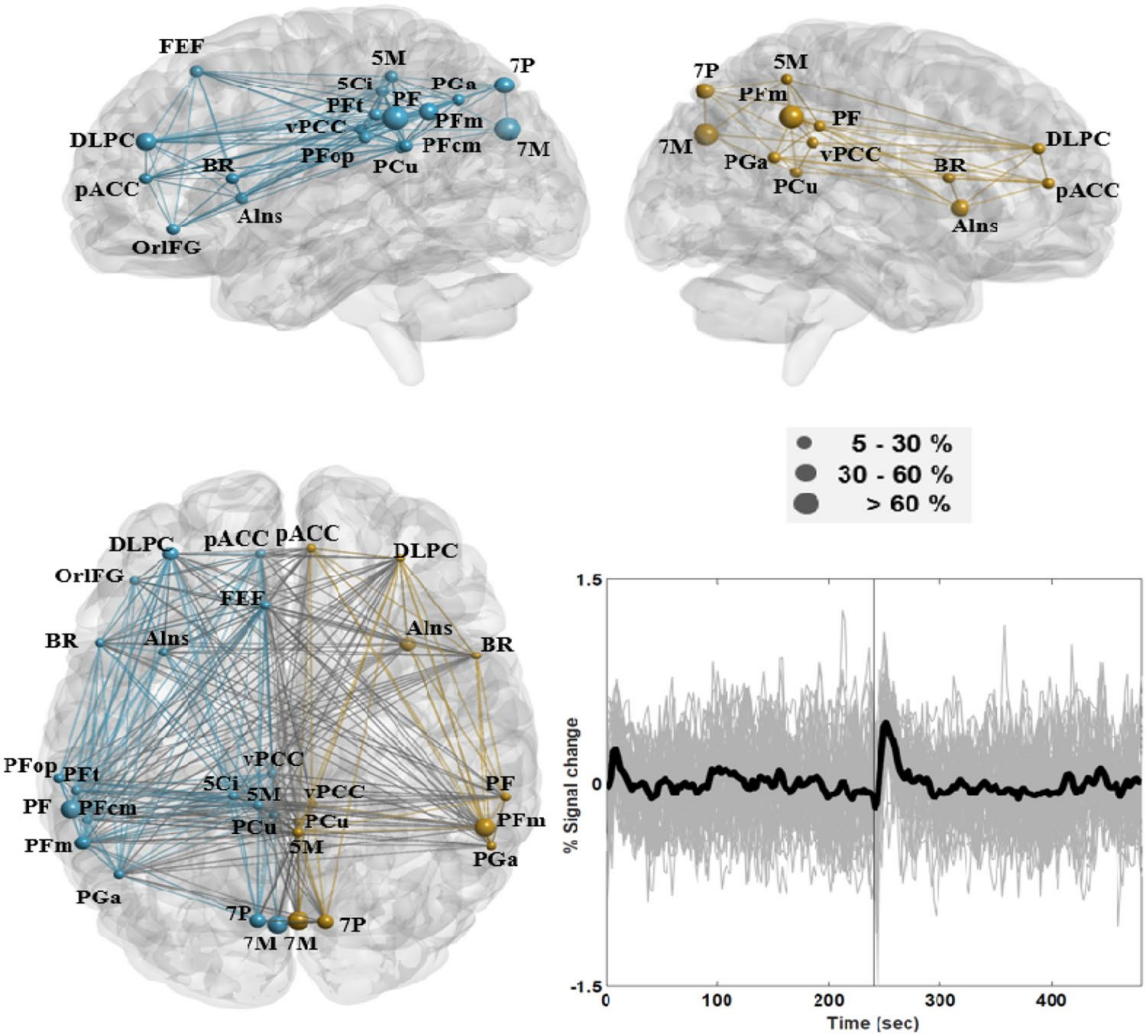
Connectivity of the transition-positive network (TPN), which engaged Broca’s region (BR), the left frontal eye field (FEF), dorsolateral prefrontal cortex (DLPC), pregenual anterior cingulate cortex (pACC), posterior cingulate cortex (vPCC and PCu), anterior angular gyrus (PGa), anterior insula (AIns), and left orbital part of inferior frontal gyrus (OrIFG). The network also engaged Areas 7P, 7M, 5Ci and 5M in the superior parietal cortex, along with Areas PF, PFm, PFcm, PFop, and PFt in the supramarginal gyrus. The nodal areas in the left hemisphere are indicated in blue, and those in the right hemisphere are indicated in gold. The diameter of the ball indicates the proportion of supra-threshold voxels in a given area of the network. The chart in the lower right panel depicts subject-level mean time courses in grey across supra-threshold voxels in the TPN with group-level mean time course shown in bold. The solid vertical line in the center indicates the onset time of the EO instruction (Color figure online)

Figure 3 depicts the connectivity network of this group of ROIs based on group-level mean time courses as revealed by BrainNet Viewer, along with plots of subject-level mean time courses in the network (Note: subject-level mean time courses were plotted for the 49 subjects who received EC-EO instructions). Connectivity values were computed using subject-level mean time courses in cytoarchitectonic areas. For example, the pACC in Table 2 includes Areas p24ab, p24c and p32, and subject-level mean time courses in these areas were separately correlated with other ROIs such as the DLPC. For the sake of simplicity, Fig. 3 depicts only the connectivity line between the pACC and DLPC. The nodal areas in the network were bilaterally distributed in the fronto-parietal areas, with more connectivity lines in the left hemisphere. According to the plots of subject-level mean time courses, this network exhibited increased activity following auditory instructions, and the amplitude as well as duration of the increased activity were higher and lasted longer after EO onset than after EC onset. Brain reactions to the EO instruction tended to be synchronized among subjects to a higher degree during the first 60-s when compared with temporal responses in other time intervals.

**Fig. 4:**
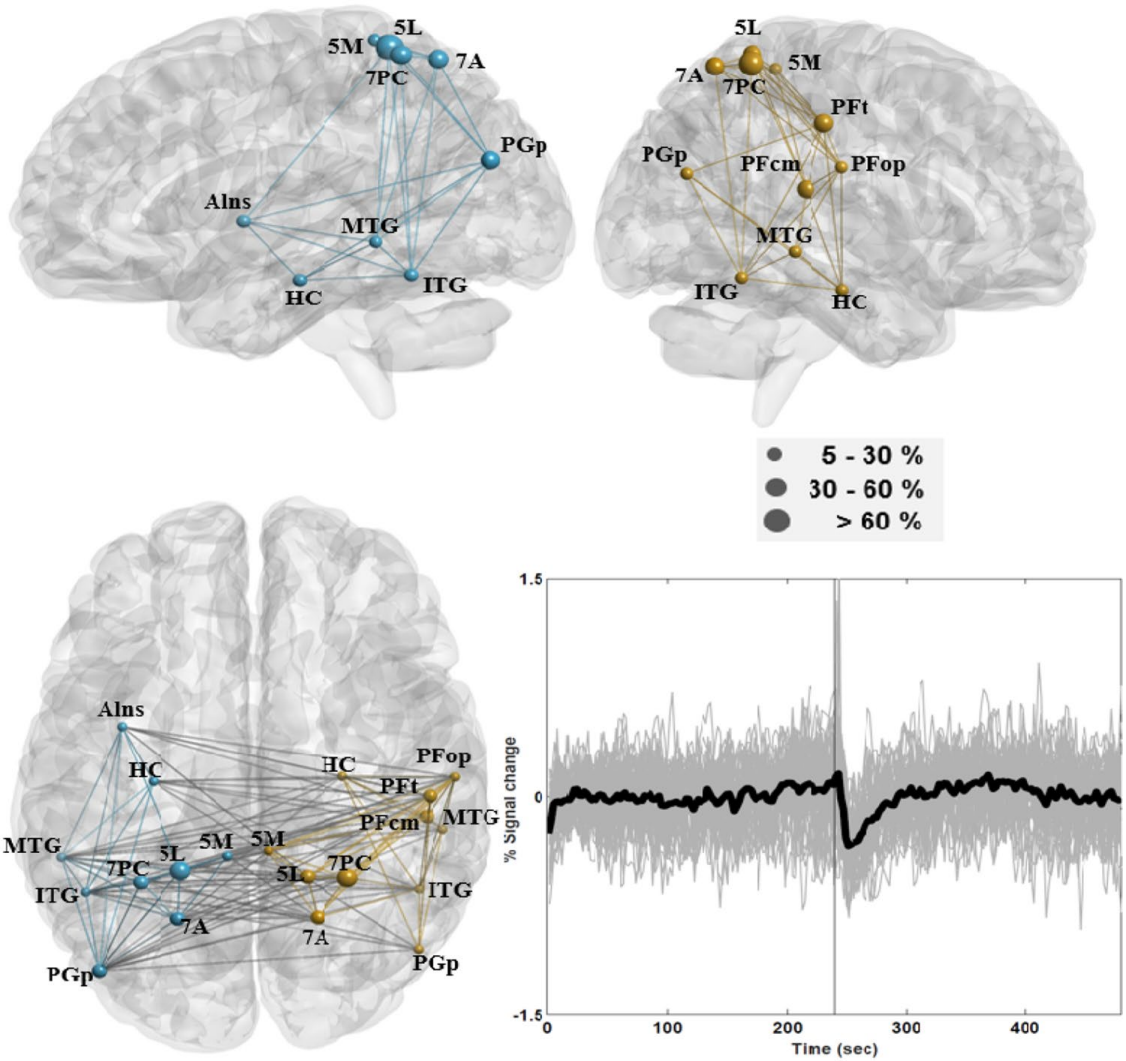
Connectivity of the transition-negative network (TNN), which engaged the hippocampus (HC), left anterior insula (AIns), inferior temporal gyrus (ITG), middle temporal gyrus (MTG), part of the superior parietal cortex (5L, 5M, 7PC, and 7A), caudal angular gyrus (PGp), and supramarginal gyrus (PFcm, PFop, and PFt in the right hemisphere). The diameter of the ball indicates the proportion of supra-threshold voxels in a given area of the network. The chart in the lower right panel depicts subject-level mean time courses in grey across supra-threshold voxels in the TNN areas with group-level mean time course indicated in bold

The second identified network exhibited decreased activity after EC or EO onset, herein referred to as the transition-negative network (TNN). ROIs within this network were the (i) hippocampus (HC; CA1-3, DG, and SC), (ii) inferior temporal gyrus (ITG), (iii) middle temporal gyrus (MTG), (iv) superior parietal cortex (5L, 5M, 7PC, and 7A; 26% supra-threshold voxels in 5M were classified within this network), (v) caudal angular gyrus (PGp), (vi) supramarginal gyrus (SG; PFcm, PFop, and PFt in the right hemisphere), and (vi) anterior insula (AIns; 28% supra-threshold voxels in AIns were classified within this network and located in the left hemisphere). ROIs within this network also had overlaps with brain areas in the DMN except for the hippocampus and anterior insula. Figure 4 depicts the connectivity network linking this group of ROIs as well as plots of subject-level mean time courses. The nodal areas in the TNN were bilaterally distributed in the temporo-parietal lobes; however, they also included areas in the right SG. Based on the subject-level mean time courses depicted in the figure, the amplitude and duration of decreased activity following auditory instructions were higher and lasted longer after EO onset than after EC onset.

**Fig. 5:**
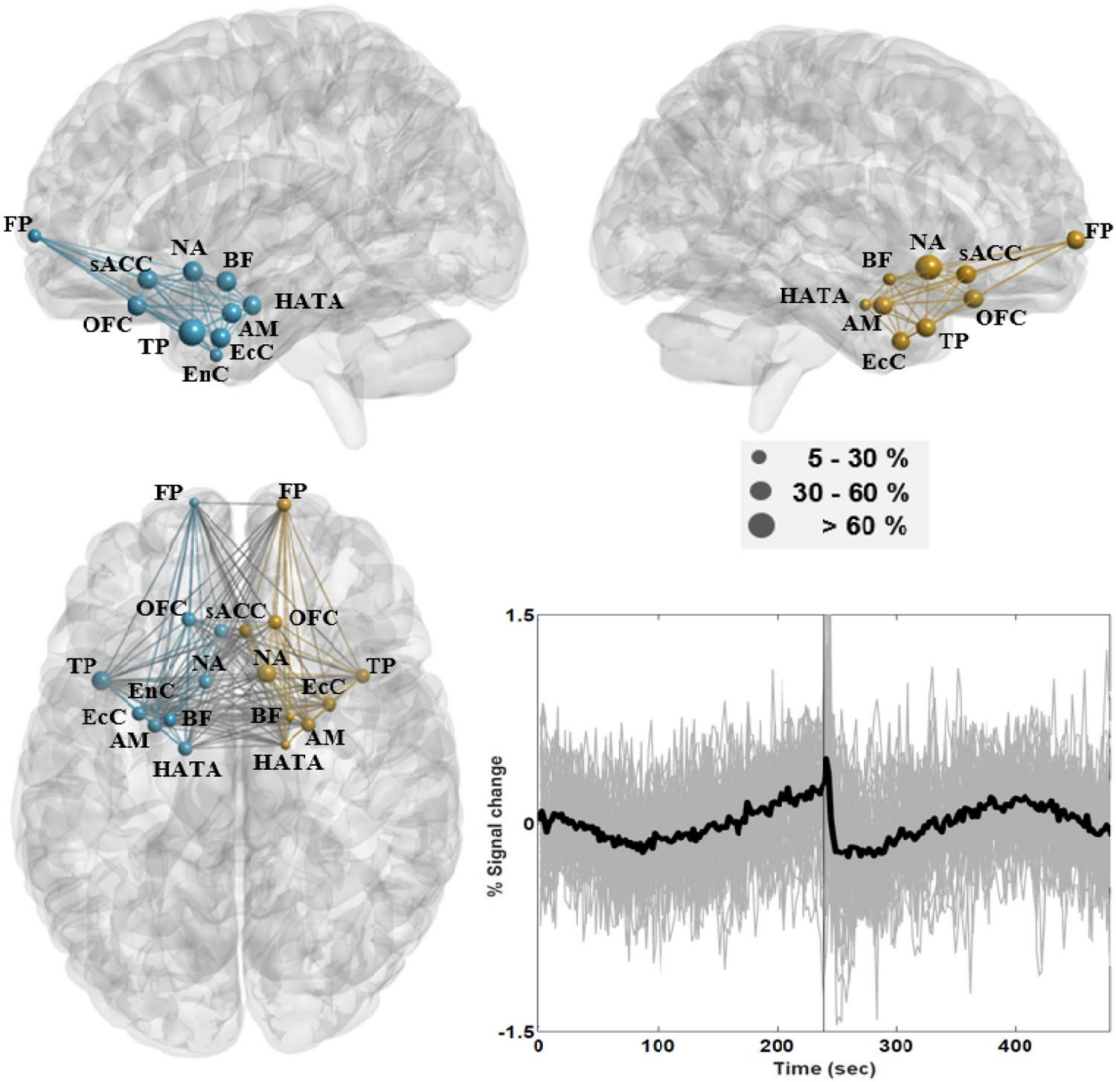
Connectivity of the stress-response network (SRN), which engaged the amygdala (AM), nucleus accumbens (NA), basal forebrain (BF), left entorhinal cortex (EnC), ectorhinal cortex (EcC), frontal pole (FP), orbito-frontal cortex (OFC), subgenual anterior cingulate cortex (sACC), temporal pole (TP), and Area HATA in the hippocampus. The nodal areas in the left hemisphere are indicated in blue, and those in the right hemisphere are indicated in gold. The diameter of the ball indicates the proportion of supra-threshold voxels in a given area of the network. The chart in the lower right panel depicts subject-level time courses in grey across supra-threshold voxels in SRN areas with the group-level mean time course indicated in bold (Color figure online)

The SRN (excluding the hippocampus) overlapped with the MPN and presented homogeneous time courses under the EC/EO conditions. The network engaged the (i) amygdala (AM; CM, SF, IF, MF, LB, VTM, and the Astr area), (ii) nucleus accumbens (NA), (iii) basal forebrain (BF; Ch1-3 and Ch4), (iv) entorhinal cortex (EnC; 11% versus 4% supra-threshold voxels in the left and right hemispheres), (v) ectorhinal cortex (EcC), (vi) frontal pole (FP; Fp1 and Fp2), (vii) orbito-frontal cortex (OFC; Fo1, Fo2, and Fo3), (viii) subgenual anterior cingulate cortex (sACC; s24, s32, 25, and 33), (ix) temporal pole (TP), along with (x) Area HATA in the hippocampus. Figure 5 depicts the connectivity network for this group of ROIs, which are distributed mainly in limbic and subcortical structures. Subject-level mean time courses in the network are also depicted in the figure. It is interesting to note that the SRN presented a prolonged decrease in activity after EC onset, and a short-term decrease with a subsequent long-term increase in activity after EO onset. The SRN was not affected by brain reactivity to the EC instruction except for a real short-term decrease in activity, whereas its activity was drastically changed after EO onset. According to the plots, it is difficult to characterize the influence of the EO instruction on time courses in the SRN, due to differences in the temporal patterns of the two conditions in response to auditory instructions.

### Association Between Salivary Cortisol and Connectivity

Table 3 lists the means and standard deviations of demographic factors and cortisol levels among the 25 subjects in the saliva group. The STAI-State anxiety and stress scores were generally higher among males than among females; however, the difference did not reach the level of statistical significance. Post-scan cortisol levels were significantly higher than pre-scan cortisol levels (p = 0.04), and the difference was contributed mainly by males. A total of 10 subjects (8 females; average age 24.60 ± 2.63) presented a decrease in cortisol levels from pre-scan (3984.64 ± 2484.33) to post-scan (3015.18 ± 1839.66), whereas the other 15 subjects (five females; average age 22.67 ± 2.19) presented increases from pre-scan (3372.56 ± 1350.26) to post-scan (6779.81 ± 4696.16). Subjects presenting an increase in cortisol levels also had higher average scores on STAI-State (37.07 vs 32.60), STAI-Trait (42.73 vs 39.70) and SQ-ISMA (11.00 vs 8.30) scales, compared to those with a decrease in cortisol levels; however, the differences did not reach the level of statistical significance. We did not observe significant differences between the two groups in pre-cortisol levels (p = 0.489); however, the two groups differed significantly in post-cortisol levels (p = 0.011). Across the entire sample, pre-cortisol levels were positively correlated with post-cortisol levels (r = 0.417, p = 0.038). After controlling for gender and age, scale scores were more strongly correlated with post-cortisol levels than with pre-cortisol levels, as follows: STAI-State scores (0.118 vs 0.021), STAI-Trait scores (0.341 vs 0.224) and SQ-ISMA scores (0.271 vs 0.073).

**Table 3:** Means and standard deviations of demographic characteristics and cortisol levels

Characteristics	Entire sample	Male	Female
Demographic			
Age	23.44	23.42	23.46
	(2.52)	(2.19)	(2.88)
STAI-state	35.28	37.17	33.54
	(11.37)	(14.79)	(7.16)
STAI-trait	41.52	41.25	41.77
	(8.17)	(8.20)	(8.47)
SQ-ISMA	9.92	10.75	9.15
	(3.79)	(3.82)	(3.74)
Neuroendocrine			
Pre-cortisol	3617.39	4142.91	3132.29
	(1863.24)	(1917.53)	(1744.05)
Post-cortisol	5273.96	6680.71	3975.42
	(4204.41)	(4650.07)	(3424.49)

Table 4 lists partial correlation values among pre/post-cortisol levels, intra-hemispheric connectivity, and inter-hemispheric connectivity within individual networks after controlling for the effects of demographic factors (Note: Age, gender, STAI-Trait and SQ_ISMA scores were included in the computation of partial correlation values; the STAI-State score did not significantly alter the correlation values in Table 4). These correlation values measured the unique contributions of connectivity values independent of the effects of demographics. Table 4 also lists the means and standard deviations of connectivity values among the 25 subjects. Connectivity values are presented as Fisher Z-transformed correlation values standardized by 1/$\sqrt{240}$ (Note: Negative values indicate negative connectivity). On average, the strength of connectivity was higher in the left hemisphere than in the right hemisphere particularly within the TNN and SRN. Partial correlation values between pre-cortisol levels and intra-hemispheric connectivity values were significant only in the SRN (p < 0.05). Partial correlation values between pre-cortisol levels and inter-hemispheric connectivity within the TNN and SRN were also significant (p < 0.05).

**Table 4:** Means and standard deviations of within-network connectivity values and partial correlation values (with p-values) between cortisol levels and connectivity values

Networks	Mean (Std)	Pre-cortisol	Post-cortisol
Intra-hemispheric			
TPN (left)	4.54 (0.96)	− .371 (.097)	− .045 (.847)
TPN (right)	4.33 (0.98)	.249 (.276)	− .133 (.566)
TNN (left)	5.06 (0.94)	.401 (.072)	.316 (.163)
TNN (right)	4.03 (0.82)	.181 (.432)	.102 (.659)
SRN (left)	4.12 (1.71)	**.545 (.011)**	.266 (.244)
SRN (right)	3.93 (2.37)	**.500 (.021)**	.274 (.229)
Inter-hemispheric			
TPN	4.02 (1.20)	.200 (.385)	− .028 (.905)
TNN	3.90 (1.26)	**.581 (.006)**	.317 (.162)
SRN	3.63 (2.01)	**.554 (.009)**	.318 (.160)

In Table 5, between-network connectivity values between the SRN and TNN were significantly positively correlated with pre-cortisol levels, despite far weaker connectivity compared with that of within-network connectivity. Overall, we observed positive between-network connectivity between the TNN and SRN, both of which presented negative connectivity with the TPN. Due to the small sample size (25 subjects), none of the partial correlation values for post-cortisol levels reached the level of statistical significance. Nonetheless, the results in Tables 4 and 5 suggest that inter-hemispheric connectivity within the TNN and SRN was sensitive to post-cortisol levels (0.317 and 0.318), whereas the between-network connectivity between the TPN and SRN was also sensitive to post-cortisol levels (− 0.313 and − 0.328). A comparison of connectivity values between Tables 4 and 5 revealed that, under the EC-EO (or EO-EC) condition, within-network connectivity was far stronger than between-network connectivity. Tables C and D in the Supplementary Materials list the within- and between-network connectivity values and corresponding correlation values with cortisol levels, respectively, under the EC and EO conditions. Note that between-network connectivity between the SRN and TNN was contributed mainly by time courses under the EC condition. In the TNN, within- and between-network connectivity was significantly correlated with pre-cortisol levels under the EC condition. In the SRN, the correlation with pre-cortisol levels was less affected by EC and EO conditions, such that correlation coefficients under the two conditions were roughly the same.

**Table 5:** Means and standard deviations of between-network connectivity values and partial correlation values (with p-values) between cortisol levels and connectivity values

Networks	Mean (Std)	Pre-cortisol	Post-cortisol
Intra-hemispheric			
TPN/TNN (left)	− 1.71 (1.37)	− .325 (.151)	− .132 (.567)
TPN/TNN (right)	− 1.20 (0.86)	− .376 (.093)	− .058 (.801)
TPN/SRN (left)	− 0.43 (1.08)	− .299 (.188)	− .313 (.167)
TPN/SRN (right)	− 0.62 (0.90)	− .275 (.228)	− .328 (.147)
TNN/SRN (left)	3.08 (1.29)	**.561 (.008)**	.301 (.184)
TNN/SRN (right)	2.39 (1.07)	**.506 (.019)**	.105 (.650)
Inter-hemispheric			
TPN (left)/TNN (right)	− 1.98 (0.82)	.068 (.769)	− .008 (.972)
TPN (left)/SRN (right)	− 0.68 (0.90)	− .153 (.508)	− .241 (.293)
TPN (right)/TNN (left)	− 2.14 (0.79)	− .231 (.314)	.039 (.866)
TPN (right)/SRN (left)	− 0.95 (1.01)	− .162 (.483)	− .223 (.332)
TNN (left)/SRN (right)	2.49 (1.49)	**.593 (.005)**	.372 (.097)
TNN (right)/SRN (left)	2.06 (1.03)	**.671 (.001)**	.237 (.302)

Correlation values with p < 0.05 are highlighted in bold

## Discussion

This study considered the strength and duration of short-term effects induced by brain reactions to closing and opening the eyes in the DMN, FPN, SN, MPN, and SRN, along with the associations between these effects and cortisol levels. We found that most ROIs in the conventional DMN, FPN, and SN were classified within the TPN, including the well-known DLPC, PCC, and SG. The effects of opening the eyes on the TPN lasted for roughly 60-s, during which temporal responses presented a short-term increase in activity, as indicated by plots of subject-level mean time courses in Fig. 3. After EC/EO onset, brain areas in the TNN overlapped with the DMN and SN and presented activity opposite to that observed in the TPN (Fig. 4). This means that when investigating resting state activity in brain areas within the TPN and TNN, images obtained during the first 60-s after EC/EO onset should perhaps be discarded to minimize potential confounding effects. Note that the hippocampus in the MPN was classified within the TNN. The prolonged decrease in activity in the SRN was minimally affected by brain reactivity to closing the eyes; however, overall activity patterns in the network presented significant changes after the onset of opening the eyes. Within the SRN, there was no simple way to evaluate the strength or duration of effects caused by opening the eyes. Moreover, the SPC (anterior vs. posterior), AG (rostral vs. caudal), and SG (left vs. right) exhibited distinct response patterns within the same areas; for example, areas in the AG classified within the TPN (PGa) and TNN (PGp). Connectivity was stronger in the left hemisphere than in the right hemisphere, and was more strongly correlated with pre-cortisol levels in the SRN than in other networks. Inter-hemispheric connectivity within the TNN and SRN was sensitive to pre-cortisol as well as post-cortisol levels. Under the EC condition, connectivity in the TNN was significantly correlated with connectivity in the SRN as well as pre-cortisol levels. Despite its sensitivity to the EO condition, the SRN was significantly correlated with pre-cortisol levels under both conditions.

### Approach-Avoidance Conflicts

Prior to the rs-fMRI scanning session, subjects were instructed to follow auditory instructions to close or open the eyes while remaining motionless and relaxed. As shown in Fig. E in the Supplementary Materials, the auditory cortex presented a sharp increase in activity, followed by a short-term decrease. The sharp increase could be partially caused by head motion (See plots in Fig. F). Previous fMRI studies have provided evidence that visual tasks lead to decreased activity in the auditory cortex (Laurienti et al. 2002; Mayhew et al. 2013). In addition to head motion, the increase in activity in Fig. E might reflect reactions to auditory instructions, which could conceivably be suppressed by having the subjects fix their eyes on a central crosshair. The amygdala and hippocampus are two major limbic regions receiving either direct or indirect neuronal inputs from the auditory cortex to support the processing of auditory working memory (Kraus and Canlon 2012; Munoz-Lopez et al. 2010). Figure G in the Supplementary Materials presents the mean time courses in supra-threshold voxels and the corresponding ICC map of the hippocampus (Areas CA1-3, DG and SC). In the figure, the anterior (ventral) part of the hippocampus (vHC) presented an immediate increase followed by a short-term decrease in activity after EO onset (Note: Pilot subjects did not clearly show similar activity after EO onset). We can also see that the effect size (i.e., decreased activity) was far larger in Area vHC than in other areas in the TNN and auditory cortex.

Auditory stimulation via the EO instruction could be viewed as novel (or aversive), due to the abruptness with which it invades the consciousness of subjects while relaxed quietly with their eyes closed. Area vHC is a common target due to its well-established role in behavior inhibition associated with resolving conflicts among competing goals (e.g., approach-avoidance conflicts). Yoshida et al. (2019) recently reported that decreased activity in Area vHC reflects the suppression of aversive and emotional states, which would otherwise compete with goal-directed or motivational behavior. In the regulation of emotional responses, Area vHC is functionally associated with limbic structures, such as the AM, NA, hypothalamus, and prefrontal cortex (Note: The hypothalamus presented activity similar to that observed in the SRN). This means that decreased activity in Area vHC could release subjects from behavioral inhibition in favor of goal-directed behavior. By contrast, fear and stress have been shown to induce rapid and dramatic alterations in the respiratory effort, cardiac output, and blood pressure (Guyenet 2006). In previous studies, stimulation of Area vHC was shown to produce sustained increases in heart rate and blood pressure in normal rats (Ajayi et al. 2018), whereas bilateral inhibition of Area vHC was shown to enhance parasympathetic responses without changes in sympathetic responses (Kuntze et al. 2016).

When reacting to novel (aversive) stimuli, the duration of an orienting response is normally shorter than that of a freezing response (specific to threatening stimuli), both of which occur prior to the fight-or-flight response and engage motor immobility as a parasympathetic brake on the otherwise active motor system (Hagenaars et al. 2014; Roelofs 2017). In the current study, we observed an immediate increase followed by a short-term decrease in activity after EO onset in the caudal premotor cortex (6d1, 6d3, and 6mc-SMA), anterior parietal cortex (1, 2, 3a, 3b), and motor cortex (4a, and 4b), echoing the responses observed in Area vHC. We hypothesize that the EO auditory instruction induced an immediate increase followed by a short-term and sharp decrease in activity in brain areas that react to auditory stimulation and participate in the suppressed fight-or-flight response aiming to sustain on-going behavior (i.e., remaining motionless with eyes fixed on a central crosshair).

### Different Types of EC/EO Effects

As shown in the plots of subject-level mean time courses in Figs. 3, 4, and 5, a high degree of synchronicity was observed after EO/EC onset and at no other times. The interpretation of the three networks below is mainly based on brain reactions to the EO instruction.

#### Transition-Positive Network

Brain areas responsible for cognitive control, attention, language processing, working memory, self-referential thought, and the detection of salient stimuli exhibited a short-term increase in activity after EO onset. Researchers have previously demonstrated that fixating on a target activates the fronto-parietal network as well as areas in the visual cortex (Ischebeck 2021). Thus, it is reasonable to hypothesize that brain areas within the TPN participate in controlling eye-movement and eye-fixation (Gonzalez et al. 2016; Mort et al. 2003; Neggers et al. 2012; Pierrot-Deseilligny et al. 2003; Pierrot-Deseilligny et al. 2004). Multiple studies have reported that the right FEF is involved in the processing of visual searches within near-field and far-field space domains (Lane et al. 2013), whereas the left FEF is responsible for short-term storage and the retrieval of information related to spatial position (Campana et al. 2007). The left FEF presented a higher proportion of supra-threshold voxels than did the right FEF, which suggests that it might be engaged in processing information related to the spatial location of the central crosshair. One study suggested that the DLPC is involved in the control of memory-guided saccades and plays a crucial role controlling eye-movement and maintaining memorized information for anticipatory saccades (Pierrot-Deseilligny et al. 2003). The PCC (vPCC and PCu) is active during reflexive saccades, but not during voluntary saccades (Carrasco 2011; Mort et al. 2003). Reflexive saccades involve visual perception (e.g., the central crosshair), which automatically engages the thalamic nuclei as well as lobules in the cerebellar vermis (Note: The thalamic nuclei and cerebellar vermis presented increased activity similar to those plotted in Fig. 3). The pACC is part of an extended medial prefrontal network, and is functionally connected to the DLPC and PCC in healthy controls under resting-state conditions (Argaman et al. 2020). The SPC is involved in saccade-related behavior responding to changes in retinal visual information related to the position and orientation of an object (Baltaretu et al. 2020). In the current study, the supra-threshold voxels in the SPC were contributed mainly by Areas 5M and 7P. It has been suggested that the AIns is involved in the target detection network, along with the SG, BR, SPC, and ACC (Ardekani et al. 2002; Clarke et al. 2000).

The right AG (PGa) plays an important role in spatial orientation and transformation, and is strongly associated with many spatial, perceptual, and cognitive processes. It is important to note that the right AG is also responsible for maintaining attention and encoding salient events occurring in the surrounding environment (Seghier 2013; Singh-Curry and Husain 2009). Generally, spatial attention lateralizes toward the right AG and semantic access lateralizes toward the left AG (Chambers et al. 2004; Seghier 2013). The inferior frontal gyrus (IFG) includes the OrIFG and BR, which perform essential functions in speech-language production. Researchers have previously shown that the anterior portion of the BR is selectively recruited for semantic processing (Area 45 had 10.6% and 11.4% supra-threshold voxels in the left and right hemispheres, respectively). By contrast, functionality of the posterior portion is specific to phonological processing during word production (Area 44 had 31.3% and 10.9% supra-threshold voxels in the left and right hemispheres, respectively) (Klaus and Hartwigsen 2019). Areas 45 and 47 in the left hemisphere have been linked to Chinese auditory lexicosemantic processing (The orIFG had 19.4% and 3.4% supra-threshold voxels in the left and right hemispheres, respectively) (Wu et al. 2009; Zou et al. 2016). Area PFm in the SG presented right hemispheric dominance, whereas other areas presented left hemispheric dominance. Neuroimaging studies have suggested that the left SG is involved in short-term memory (in linguistic phonology) and/or in auditory working memory (in general) (Becker et al. 1999). The left SG is specifically linked to pitch memory performance, whereas the right SG appears to be involved in rhythm memory (Schaal et al. 2017). The activation of the IFG and SG may also depend on the content of the spoken language (i.e., auditory instructions in Chinese). To summarize, the TPN formed a connectivity network that appeared to be involved in controlling eye-movement and detecting the central crosshair. The TPN also reflected brain reactivity to phonological components in auditory instructions spoken in Chinese.

#### Transition-Negative Network

Brain areas within the TNN are distributed along the classical ventral and ventro-dorsal streams of the visual system (Faillenot et al. 1997; Hebart and Hesselmann 2012; Rizzolatti and Matelli 2003; Ungerleider 1982). In the current study, this network presented a short-term decrease in activity, particularly after EO onset. It has been established that the ventral stream is involved in categorizing visual objects lateralized to the left hemisphere and storing the attributes of objects (e.g., faces) lateralized to the right hemisphere. This pathway includes the lateral temporal cortex (ITG and MTG), BA19 and the fusiform gyrus (FG). Cytoarchitectonic areas within BA19 and FG presented positive as well as negative responses after EC/EO onset, with the exception of a relatively high proportion of supra-threshold voxels that presented decreased activity. Note however that decreased activity in cytoarchitectonic areas of the FG were localized primarily to the left hemisphere. The fact that the EC/EO protocol adopted in the current study did not require the recognition or categorization of visual objects might have inhibited activity in areas within the ventral stream. The ventro-dorsal stream includes Area hOc5 and the inferior parietal cortex (Rizzolatti and Matelli 2003), which play roles in spatial and action-specific perception. Area hOc5 (corresponding to V5/MT) plays a key role in retaining information related to the direction of motions and to a lesser degree in spatial positioning. Unlike other areas of visual cognition, time courses in Area hOc5 only presented decreased activity after EO onset. As shown in Fig. 4, Areas PFcm, PFop, PFt in the SG presented right hemispheric dominance within the TNN, whereas Area PGp in the AG presented left hemispheric dominance. The left SG is engaged when naming spatial relationships between two concrete objects, whereas the right SG is engaged when a spatial task involves abstract shapes (Damasio et al. 2001). The left AG is responsible for processing semantic meaning as well as the integration of spatial information with conceptual knowledge (Hirnstein et al. 2011).

The SPC has been implicated in the provision of visual guidance associated with reaching (Battaglia-Mayer et al. 2003). When reacting to task demands, the dorso-rostral portion of the SPC operates in conjunction with Areas 7A and 7PC in combining eye and hand signals in a congruent manner within a spatial framework (Battaglia-Mayer and Caminiti 2018; Magri et al. 2019). The SPC is heavily interconnected with the dorsal premotor cortex (Wise et al. 1997), which is important in response selection, stimulus–response association, and response preparation (Passingham 1993). Function in the left AIns is specific to the perception of visual emotional stimuli (Caria et al. 2010). As mentioned previously, Area vHC initiated the orienting response to novel (aversive) stimuli, thereby engaging motor immobility as a parasympathetic brake on the otherwise active motor system to sustain goal-directed behavior. To summarize, it appears that auditory instructions could decrease activity in brain areas that were functionally unrelated to controlling eye-movement. Moreover, decreased activity in Area vHC and the motor system might be an indication of a suppressed fight-or-flight response with the aim of sustaining the on-going resting-state.

#### Stress-Response Network

One recent review in which animal models were combined with diffusion imaging tractography proposed a revision to the conventional limbic model, which treats the anterior temporal cortex (AM and TP) and OFC as a network functionally associated with behavioral inhibition (Catani et al. 2013). In the theory of behavioral inhibition proposed by Gary and Naughton, the septal nucleus (Note: Nucleus Ch1 in the BF is the medial septal nucleus) and hippocampus play critical roles in monitoring approach-avoidance conflicts. In that schema, activation of the HC constitutes non-anxious rumination, activation of the AM constitutes pure fear, and activation of both constitutes anxiety (McNaughton and Gray 2000). Thus, the AM is strongly engaged in increasing arousal when subjects encounter fear potentiated startle. Neuroimaging and post-mortem histological studies on patients suffering from chronic anxiety and depression have also revealed stress-induced changes in brain structures, including the OFC, sACC, HT, insular cortex, and EnC (Cerqueira et al. 2008; Lucassen et al. 2014). The insular cortex is commonly included as part of the stress-response system (especially the anterior insula) (Bogdan et al. 2016; Hofman and Falk 2012; Nieuwenhuys 2011); however, time courses obtained from the anterior insula in this study are suggestive of two processes: One associated with the TPN and the other associated with the TNN. Generally, brain areas in the SRN overlap considerably with the MPN, which deals with information related to emotion processing and introspection.

In the current study, the SRN exhibited a prolonged decrease in activity following the eyes-closed instruction, and a short-term decrease succeeded by a long-term increase in activity following the eyes-open instruction. Limbic deactivation has been interpreted as the suppression of unwanted emotional reactions that are aroused by tasks (Dagher et al. 2009; Dedovic et al. 2009; Lederbogen et al. 2011; Pruessner et al. 2008; Soliman et al. 2011), or conversely as full relaxation responses under resting states (Kalyani et al. 2011; Shetkar et al. 2019). As mentioned previously, a short-term decrease in the vHC was an indication that the subject was under parasympathetic control and free from approach-avoidance conflicts. Thus, it would be reasonable to hypothesize that while the subjects were relaxed under the EC condition, the SRN played a role in monitoring the external world using sensory information. A prolonged decrease in activity when the subject became accustomed to the external environment might be an indication of fMRI repetition suppression (Barron et al. 2016; Bunzeck and Thiel 2016; Grill-Spector et al. 2006). In other words, the prolonged decrease in activity might be an indication of reduced connectivity between the network and cortical structures (Barron et al. 2016; Kuo et al. 2019). Researchers have previously demonstrated that goal-directed eye movements can activate a dorsal fronto-parietal network (i.e., TPN) and transiently deactivate the amygdala (i.e., SRN) via a ventromedial prefrontal pathway to enable the cognitive regulation of emotions (de Voogd et al. 2018). These findings are consistent with our observation of time courses within the SRN under the EO condition. To summarize, the SRN presented reduced connectivity to cortical structures under the EC condition. It is possible that when the novel (aversive) stimulus was non-threatening, the observed short-term decrease succeeded by a long-term increase in activity under the EO condition was mediated by either the ventromedial prefrontal pathway or Area vHC (Patrick et al. 2019).

### Pre-scan and Post-scan Cortisol Levels

Cortisol is a catabolic hormone in normal life and an adaptation hormone in response to stressful situations (Coenen and Flik 2017). Cortisol acts on low-affinity glucocorticoid receptors (GRs) as well as high-affinity mineralocorticoid receptors (MRs). GRs are widely distributed throughout the brain with high concentrations in the hypothalamic corticotrophin-releasing-hormone neurons and pituitary corticotropes and, by contrast, the distribution of MRs is highly restricted (Andersen et al. 2013; de Kloet and Joëls 2020; Jelić et al. 2005). Both GRs and MRs are highly expressed in limbic structures, such as the hippocampus, amygdala, and brain areas associated with fear and anxiety (De Kloet et al. 2018; Deuter et al. 2019). In the current study, average cortisol levels in saliva collected prior to the fMRI scanning sessions were significantly lower than in saliva collected after scanning. Connectivity values in the three networks were also more strongly associated with pre-cortisol levels than with post-cortisol levels. As mentioned, subjects were passively exposed to 20-min of natural sound stimulation (human voices and animal vocalizations) and speech (same sex-marriage controversy) following the completion of the resting-state scan. The STAI-Trait and SQ-ISMA scores were more strongly correlated with post-cortisol levels than with pre-cortisol levels. In other words, trait-anxiety or stress scores could be predictive of post-cortisol levels.

The results in Tables 4 and 5 show that after controlling for demographic factors, connectivity within the SRN was significantly positively correlated with pre-cortisol levels. Inter-hemispheric connectivity within the SRN and TNN was also significantly correlated with pre-cortisol levels. These results are in good agreement with the high concentrations of GRs and MRs in limbic structures. As mentioned previously, ROIs in the SRN were stable loci for probing cortisol activity, despite their sensitivity to the EO instruction while the subjects were resting quietly. The strong connection between the TNN and SRN under the EC condition might account for the significant correlation between connectivity in the TNN and pre-cortisol levels. It is also possible that under the EC condition, connectivity within and between the SRN and TNN could serve as a predictor of post-cortisol levels. The EC protocol is generally well-suited to research linking resting-state connectivity and GR/MR concentration in limbic structures, considering the weakness of brain reactions to the anticipated EC instruction. Our empirical data collected from one cycle of EC-EO resting state scanning did not support the hypothesis that decreased activity in the ventral hippocampus is regulated by cortisol activity, as indicated by the fact that the TNN was unaffected by pre-cortisol levels under the EO condition. Moreover, the correlation values listed in Tables 4, 5, C and D do not necessarily provide evidence supporting the role played by pre-cortisol levels in regulating EC/EO effects. As mentioned, subject-level mean time courses showed a high degree of synchronicity after EC/EO onset and at no other times. This would be a reason that we found a significant correlation between connectivity values and pre-cortisol levels within the SRN. Therefore, the ICC method provided ample information specific to individual subjects when searching for synchronized brain reactions to EC/EO instructions.

### Remarks

Rs-fMRI studies have previously used 0.01 Hz (or 0.008 Hz) as a standard cutoff to remove low frequency effects such as slow drifts in the fMRI signal (Wang 2021). Physiological noise from respiratory and cardiac functions is concentrated at higher frequencies (> 0.1 Hz) (Davey et al. 2013; Mascali et al. 2021). Thus, RSNs of interest are generally associated with frequencies in the range of 0.01–0.1 Hz. Fig. H in the Supplementary Materials plots supra-threshold time courses of the 49 subjects (receiving EC-EO instructions) after either high-pass (i.e., > 0.01 Hz) or bandpass (0.01–0.1 Hz) filtering. Note that the supra-threshold time courses were extracted using the ICC method based on pre-processed data that had not undergone filtering. In a comparison of Figs. 3, 4, 5 and H, it appears that the TPN was less affected by data filtering than were the TNN and SRN. Figures H-(b), H-(c), H-(bʹ) and H-(cʹ) further suggest that ROIs in the TNN and SRN after data filtering could be grouped into one cluster, such that activity in the ventral hippocampus and other motor areas would be indifferentiable from that in the SRN. The main hypothesis generated from our findings was the approach-avoidance conflicts which distinguished activity in the hippocampus (the transition negative network; TNN) from that in limbic structures (the stress-response network; SRN). The two networks were highly associated with each other under the EC condition, but disconnected under the EO condition. As mentioned, the SRN is significantly associated with pre-cortisol levels under both EC and EO conditions, but the TNN is significantly associated with pre-cortisol levels only under the EC condition. The filtering procedure (especially high-pass filtering) in the pre-processing stage could affect the scientific interpretation of TNN and SRN.

Changes in heart-rate and respiration could have confounding effects on rs-fMRI time courses. Because electrocardiograms (ECGs) and breathing patterns were not collected during resting-state scans, it is difficult to provide a concise assessment of these effects. However, high-pass and bandpass filtering provided similar results as indicated in Fig. H. It appears that heart-rate and respiration might not necessarily have a serious confounding effect on the analysis of brain reactions to closing/opening the eyes. Head motion after EC/EO onset could also affect the assessment of synchronicity in the ICC method. Values of FD and DVARS across the time scale are presented in Fig. F in the Supplementary Materials for the 49 subjects receiving EC-EO instructions. These values were computed using realigned image volumes within individual subjects before the stages of removing trends and normalization to the MNI template. There were two subjects who did show larger FD values after EC/EO onset compared with other subjects. In Fig. F, the large mean values after EC/EO onset (especially after EC onset) mainly reflect head motion of these two highlighted subjects (e.g., See blue and red curves). Because the ICC method considers synchronicity across all subjects, we assume that head motion of a few subjects after EC/EO onset had minor effects on interpretation of the three networks.

### Limitations

The order of EC and EO instructions had minimum effects on the TPN, but had significant effects on the TNN and SRN according to brain reactions to auditory instructions in the 6 pilot subjects. However, the order of instructions did not show significant effects on the assessment of correlation between connectivity and cortisol levels. Our experiment was limited to one cycle of the EC-EO transition, and findings in the Results section could partially reflect a variety of diverse processes such as listening to instructions, opening the eyes, and novelty responses. The experimental design did not allow to disentangle the specific contributions of these processes to the observed data. A further study could adopt multiple cycles of the EC-EO transitions to investigate effects from different processes (Feige et al. 2005). Budget considerations limited us to 25 assays for cortisol levels (including cortisol levels in the 6 pilot subjects). Given the small sample size in the current study, we were unable to provide a reliable test on the hypothesis of approach-avoidance conflicts as regulated by Area vHC (or alternatively by a ventromedial prefrontal pathway).

## Conclusion

The study is the first attempt to connect cytoarchitectonically defined brain structures to brain reactivity patterns associated with closing and opening the eyes under resting-state conditions. We recommend that research based on rs-fMRI adopts the EC condition when probing resting-state connectivity and its neuroendocrine correlates. We also suggest that abrupt instructions to open the eyes (while subjects are resting quietly with EC) could be used as a probe to characterize brain reactivity to aversive stimuli in the ventral hippocampus and other limbic structures. Finally, we encourage further work in this direction, particularly from a clinical perspective.

## Supplementary Information

Below is the link to the electronic supplementary material.

Supplementary file1 (DOCX 1943 kb)

## Data Availability

FMRI raw data are available upon request to the corresponding author.

## Footnotes

1 Among the 55 subjects, 49 of them received the EC-EO instructions and the 6 pilot subjects received the EO-EC instructions. Suprathreshold voxels masked out by the ICC method were found by the evaluation of synchronicity among the 49 subjects (who followed the same order of instructions). The spatial locations of these voxels were applied to extract time courses of the 6 pilot subjects. The evaluation of statistical assumptions underlying the ICC method and the connectivity study were applied to the 55 subjects. The pilot subjects were interviewed on their experiences of the experimental procedures after fMRI scanning.
