# Supplementary material for: Brain Reactions to Opening and Closing the Eyes: Salivary Cortisol and Functional Connectivity
Source: Brain Topogr. 2022 Jun 6;35(4):375–97. doi: 10.1007/s10548-022-00897-x (PMC9334428; doi:10.1007/s10548-022-00897-x)
Supplement: Supplementary file 1 — Supplementary file1 (DOCX 1943 kb) [file 10548_2022_897_MOESM1_ESM.docx]

**Supplementary Materials**

**A. The asymptotic results of the ICC(A,M) estimate**

We use the analysis of variance (ANOVA) notations given in McGrow and Wong (1996) to derive $\hat{A}$ in (1) (Note: M denotes the number of subjects and n denotes the length of each fMRI time course)

| Table A: ANOVA notations for deriving $\hat{C}$ and $\hat{A}$ indices. | | | |
| --- | --- | --- | --- |
| Sources of variation | Sum squares | Degrees of freedom | Mean squares |
| Between Rows (times) | SS_R_ = $\frac{n}{M}$(${\underline{\boldsymbol{1}}}^{'}\mathbf{S}\underline{\mathbf{1}}\boldsymbol{)}$ | n-1 | MS_R_ =$\frac{n}{M(n-1)}$ (${\underline{\boldsymbol{1}}}^{'}\mathbf{S}\underline{\mathbf{1}}\boldsymbol{)}$ |
| Between columns (subjects) | SS_C_ = $\frac{M}{n}$(${\underline{\boldsymbol{1}}}^{'}\boldsymbol{V}\underline{\mathbf{1}}\boldsymbol{)}$ | M-1 | MS_C_ = $\frac{M}{n(M-1)}$ (${\underline{\boldsymbol{1}}}^{'}\boldsymbol{V}\underline{\mathbf{1}}\boldsymbol{)}$ |
| Rows by columns interaction | SS_RxC_ = n[**tr(S)**] – $\frac{n}{M}$ (${\underline{\boldsymbol{1}}}^{\boldsymbol{'}}\boldsymbol{S}\underline{\mathbf{1}}\boldsymbol{)}$ | (n-1)(M-1) | MS_CxR_ = $\frac{1}{M(n-1)(M-1)}\left\{ Mn[\mathbf{tr(S)}] - n({\underline{\boldsymbol{1}}}^{'}\boldsymbol{S}\underline{\mathbf{1}}\boldsymbol{)} \right\}$ |

In Table A, $\mathbf{S}$ is the M-by-M cross-covariance matrix with zero lagged time points among preprocessed fMRI time courses within a single voxel. It is a column-wise covariance matrix that measures the variation of average intensity across time points. The row-wise n-by-n covariance matrix ***V*** measures the variation of average intensity across subjects. Under the random effect model (both subjects and time points are random), the ICC index measuring consistency among subjects is estimated by

$\hat{C}$ = (MS_R_ – MS_RxC_)∕ MS_R_

= 1- (MS_RxC_∕MS_R_)

= 1 – $\left\{ M[tr(\mathbf{S})] - ({\underline{\boldsymbol{1}}}^{'}\boldsymbol{S}\underline{\mathbf{1}}\boldsymbol{)} \right\}$∕$\left[ (M-1)({\underline{\boldsymbol{1}}}^{'}\boldsymbol{S}\underline{\mathbf{1}}\boldsymbol{)} \right]$

= $\frac{M}{M-1}\left\{ 1-\left[ \mathrm{tr}\left( \mathbf{S} \right)/({\underline{\boldsymbol{1}}}^{'}\boldsymbol{S}\underline{\mathbf{1}}\boldsymbol{)} \right] \right\}$ (A.1)

and the agreement index is estimated by

$\hat{A}$ = (MS_R_ – MS_RxC_)∕ [MS_R_ + (MS_C_ - MS_RxC_)/n]

= $\hat{C}/$[1 + (MS_C_MS_RxC_)∕(nMS_R_)]. (A.2)

According to the estimates in Table A,

$\left( \frac{1}{n} \right)$ (MS_C_∕MS_R_) = $\left( \frac{M^{2}(n-1)}{n^{3}(M-1)} \right)\left[ (\underline{1}^{'}\boldsymbol{V}\underline{1})/(\underline{1}^{'}\boldsymbol{S}\underline{1}) \right]$

= $\left( \frac{(n-1)}{n(M-1)} \right)\Gamma$ (A.3)

where Γ is the ratio between $(\underline{1}^{'}\boldsymbol{V}\underline{1})/n^{2}$and $(\underline{1}^{'}\boldsymbol{S}\underline{1})$∕$M^{2}$. Also,

$-\left( \frac{1}{n} \right)$ (MS_RXC_∕MS_R_) = − $\left( \frac{1}{n} \right)\left\{ M[tr(\mathbf{S})] - ({\underline{\boldsymbol{1}}}^{'}\boldsymbol{S}\underline{\mathbf{1}}\boldsymbol{)} \right\}$∕$\left[ (M-1)({\underline{\boldsymbol{1}}}^{'}\boldsymbol{S}\underline{\mathbf{1}}\boldsymbol{)} \right]$

= $-$ $\left( \frac{M}{n(M-1)} \right)\left\{ \left[ \mathrm{tr}\left( \mathbf{S} \right) \right]/({\underline{\boldsymbol{1}}}^{'}\boldsymbol{S}\underline{\mathbf{1}}\boldsymbol{)} \right\}$ $+\left( \frac{1}{n(M-1)} \right)$

= $-\left( \frac{1}{n(M-1)} \right)$ Ψ $+\left( \frac{1}{n(M-1)} \right)$ (A.4)

≃ *O*$\left( \frac{1}{\mathrm{nM}} \right)$

where Ψ is the ratio between $\left[ \mathrm{tr}\left( \mathbf{S} \right) \right]/M$ and $(\underline{1}^{'}\boldsymbol{S}\underline{1})$∕$M^{2}$. By law of large numbers, Ψ converges to a constant value. Based on (A.3) and (A.4), it can be shown that

$\hat{A}$ = $\hat{C}/\left\{ 1+ \left( \frac{1}{n} \right)\left[ (\mathrm{MS}_{c}- \mathrm{MS}_{\mathrm{RxC}})/\mathrm{MS}_{R} \right] \right\}$*.*

= $\hat{C}/\left\{ 1+ \left( \frac{(n-1)}{n(M-1)} \right)\Gamma-\left( \frac{M}{n\left( M-1 \right)} \right)\left[ \mathrm{tr}\left( \mathbf{S} \right)/({\underline{\boldsymbol{1}}}^{'}\boldsymbol{S}\underline{\mathbf{1}}\boldsymbol{)} \right]+ \frac{1}{n(M-1)} \right\}$ (A.5)

= $\hat{C}/\left\{ 1+ \left( \frac{1}{(M-1)} \right)\Gamma+ O\left( \frac{1}{\mathrm{nM}} \right) \right\}$

≃ $\hat{C}\left[ \frac{M-1}{\left( M-1 \right)+\Gamma} \right]$ (A.6)

As n or M becomes large, the remainder term *O*($\frac{1}{\mathrm{nM}}$) converges in probability to zero. According to (A.6), the size of $\hat{A}$ has a smaller range than that of $\hat{C}$. In practical applications, both (A.5) and (A.6) are valid for estimating the ICC(A,M) index.


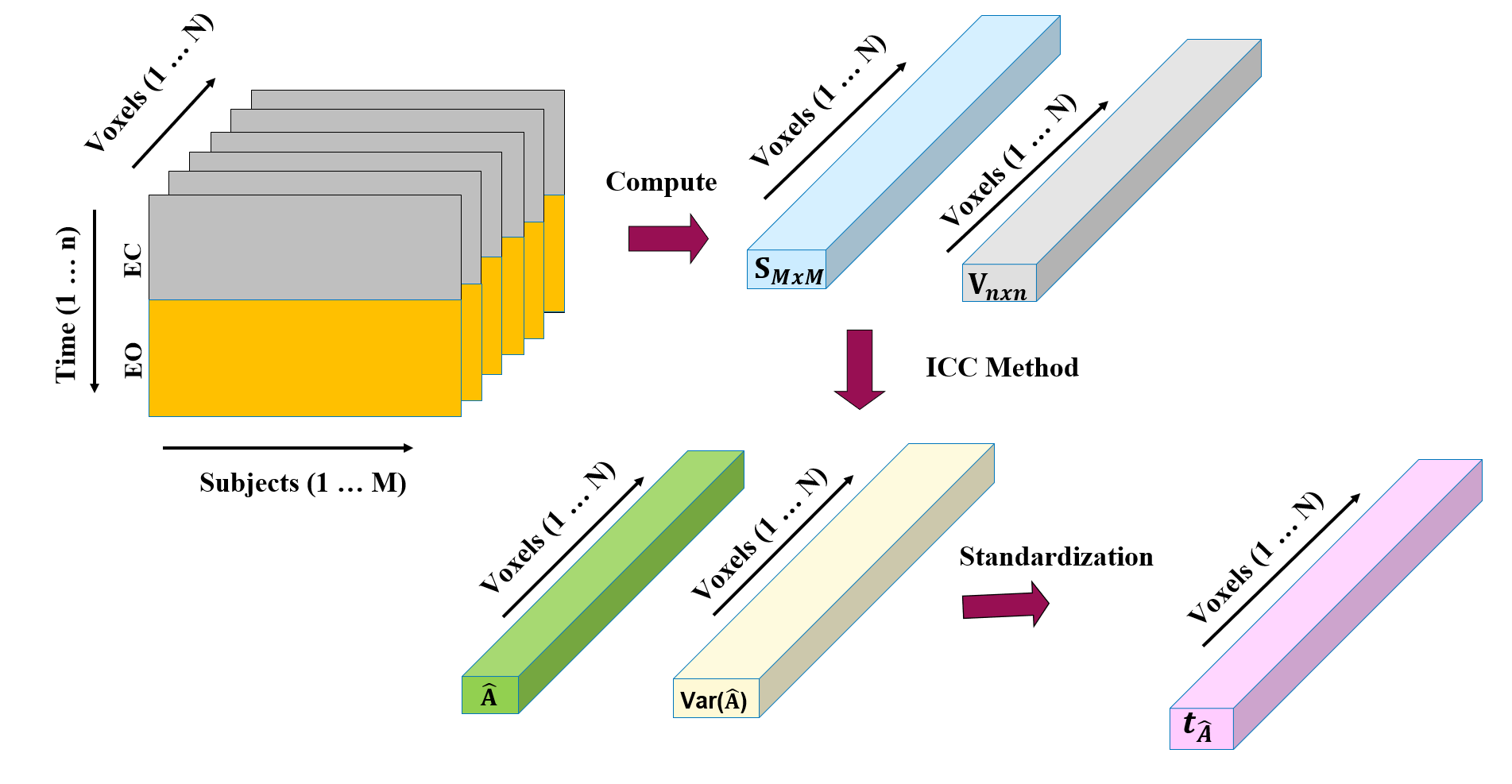


Fig. A-1: A schematic illustration of computing agreement indices and their standard errors. The input data are the pre-processed subject-by-time-by-voxel matrix. The matlab programs compute the $S_{MxM}$ and $V_{nxn}$ symmetric matrices within each voxel, where M is the number of subjects and n is the length of each fMRI time course. Based on the matrices, the ICC method generates the estimated agreement index and its error variance within each voxel. The outputs are the standardized agreement indices for all voxels. The matlab programs are available upon request to the first and corresponding authors.


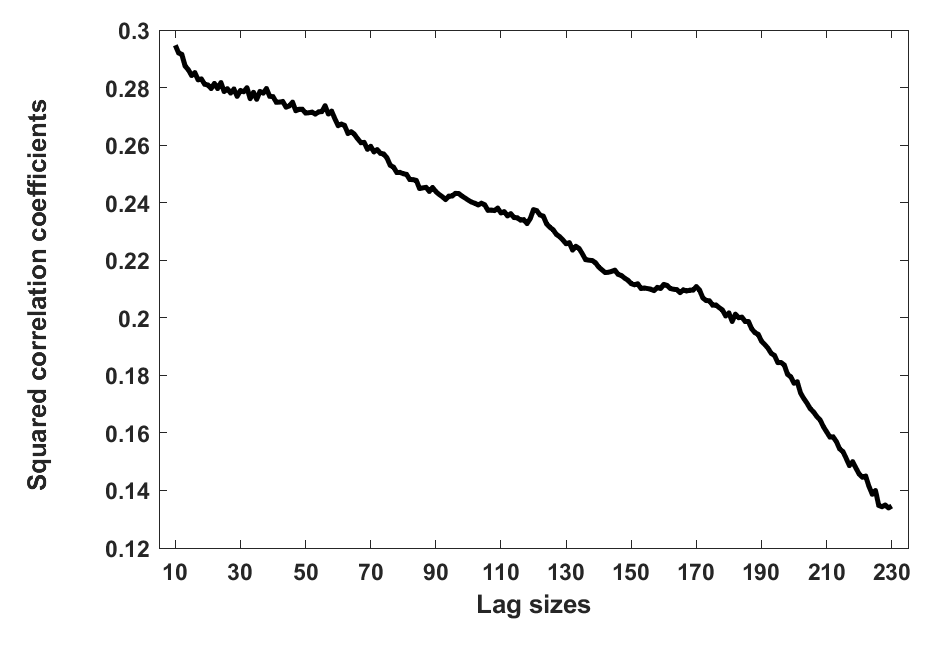


Fig. A-2: The maximal squared lagged-correlation coefficients averaged across all in-brain voxels. For sample estimates $\left\{ X_{k,i}: 1 \leq k \leq n, 1 \leq i \leq M \right\}$ with n image scans and M sessions, the squared modulus of lagged-correlation coefficients (along the time courses) is: $\max_{1\leq k<n-\upsilon,1\leq i<M}\left( \hat{\rho}\left( X_{k,i},X_{k+\upsilon, i} \right) \right)^{2}$where υ is the lag size and $\hat{\rho}$ is the estimated lagged-correlation coefficient. For each lag size and 55 available subjects, the lagged-correlation coefficients were computed for all possible pairs (*k*, *k* +$\upsilon$) and the maximum was identified within these pairs.

**B. ICC maps**

**
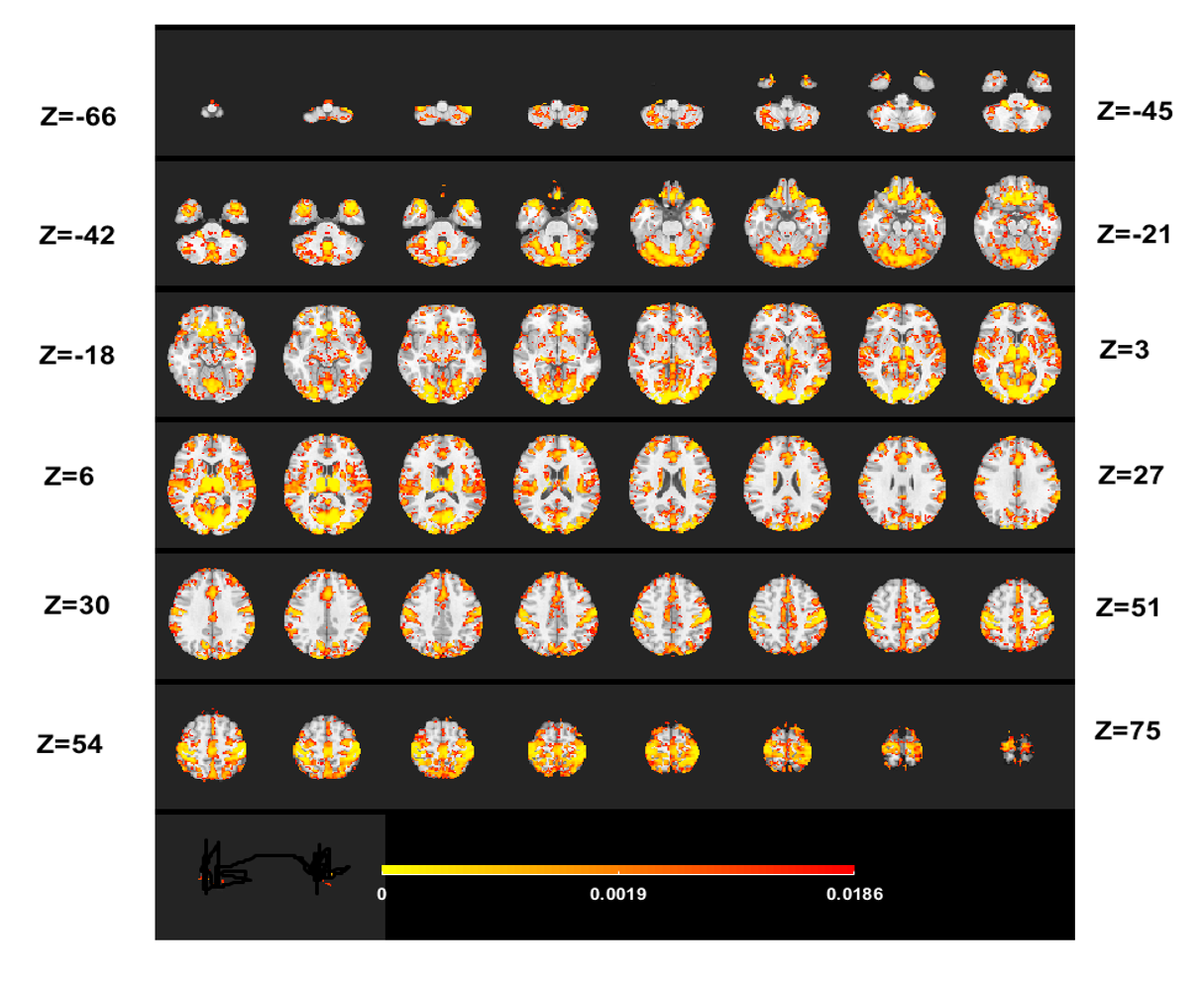
**

Fig. B: ICC maps in different axial slices presented in radiological orientation with the left hemisphere shown on the right. The supra-threshold voxels presented significant $t_{\hat{A}}$ values after applying FDR control over the false positive rate at α = 0.05. Standardized MNI Z coordinates are presented in the first and last columns in the maps (3-mm inter-slice thickness).

**C. Within- and between-network connectivity values under the eyes-closed condition and their partial correlation values with cortisol levels**

| Table C-1: Means and standard deviations of within-network connectivity values under the eyes-closed condition among subjects and partial correlations values (with p-values) between cortisol levels and connectivity values by controlling for age, gender, trait-anxiety, and stress (Note: p-values < .05 are highlighted in bold). | | | |
| --- | --- | --- | --- |
| **Networks** | **Mean (Std)** | **Pre-Cortisol** | **Post-Cortisol** |
| **Intra-hemispheric** | | | |
| TPN (left) | 3.24 (0.85) | -.338 (.134) | -.054 (.815) |
| TPN (right) | 3.24 (0.79) | .188 (.413) | -.144 (.534) |
| TNN (left) | 3.59 (0.65) | **.468 (.032)** | -.014 (.953) |
| TNN (right) | 2.92 (0.63) | **.576 (.006)** | .337 (.135) |
| SRN (left) | 2.99 (1.63) | **.508 (.019)** | .209 (.364) |
| SRN (right) | 2.83 (2.07) | **.460 (.036)** | .209 (.362) |
| **Inter-hemispheric** |  |  |  |
| TPN | 2.89 (0.88) | .077 (.740) | -.122 (.628) |
| TNN | 2.78 (0.99) | **.671 (.001)** | .304 (.180) |
| SRN | 2.66 (1.86) | **.486 (.026)** | .261 (.253) |

| Table C-2: Means and standard deviations of between-network connectivity values under the eyes-closed condition among subjects and partial correlation values (with p-values) between cortisol levels and connectivity values by controlling for age, gender, trait-anxiety, and stress (Note: p-values < .05 are highlighted in bold). | | | |
| --- | --- | --- | --- |
| **Networks** | **Mean (Std)** | **Pre-Cortisol** | **Post-Cortisol** |
| **Intra-Hemispheric** | | | |
| TPN/TNN (left) | -1.00 (0.92) | -.343 (.128) | -.040 (.865) |
| TPN/TNN (right) | -0.76 (0.59) | **-.476 (.029)** | -.020 (.930) |
| TPN/SRN (left) | -0.14 (0.79) | -.263 (.249) | -.303 (.182) |
| TPN/SRN (right) | -0.25 (0.82) | -.151 (.513) | -.232 (.311) |
| TNN/SRN (left) | 2.23 (1.20) | **.529 (.014)** | .222 (.334) |
| TNN/SRN (right) | 1.75 (0.93) | **.600 (.004)** | .177 (.442) |
| **Inter-Hemispheric** |  |  |  |
| TPN (left)/TNN (right) | -1.28 (0.61) | -.195 (.398) | .011 (.962) |
| TPN (left)/SRN (right) | -0.39 (0.80) | -.214 (.352) | -.242 (.290) |
| TPN (right)/TNN (left) | -1.35 (0.66) | -.244 (.287) | .233 (.309) |
| TPN (right)/SRN (left) | -0.56 (0.79) | -.237 (.301) | -.235 (.305) |
| TNN (left)/SRN (right) | 1.82 (1.31) | **.594 (.005)** | .343 (.128) |
| TNN (right)/SRN (left) | 1.50 (0.83) | **.645 (.002)** | .222 (.334) |

**D. Within- and between-network connectivity values under the eyes-open condition and their partial correlation values with cortisol levels**

| Table D-1: Means and standard deviations of within-network connectivity values under the eyes-open condition among subjects and partial correlation values (with p-values) between cortisol levels and connectivity values by controlling for age, gender, trait-anxiety, and stress (Note: p-values < .05 are highlighted in bold). | | | |
| --- | --- | --- | --- |
| **Networks** | **Mean (Std)** | **Pre-Cortisol** | **Post-Cortisol** |
| **Intra-hemispheric** | | | |
| TPN (left) | 3.34 (0.69) | -.301 (.185) | -.058 (.804) |
| TPN (right) | 3.04 (0.79) | .268 (.239) | -.105 (.650) |
| TNN (left) | 3.80 (1.06) | .140 (.544) | .374 (.095) |
| TNN (right) | 3.02 (0.82) | -.085 (.715) | -.057 (.806) |
| SRN (left) | 2.85 (1.05) | **.505 (.020)** | .277 (.224) |
| SRN (right) | 2.69 (1.44) | **.465 (.034)** | .267 (.242) |
| **Inter-hemispheric** |  |  |  |
| TPN | 2.89 (0.92) | .296(.193) | .003 (.989) |
| TNN | 2.80 (1.00) | .383(.087) | .278 (.222) |
| SRN | 2.49 (1.18) | **.548(.010)** | .303 (.182) |

| Table D-2: Means and standard deviations of between-network connectivity values under the eyes-open condition among subjects and partial correlation values (with p-values) between cortisol levels and connectivity values by controlling for age, gender, trait-anxiety, and stress (Note: p-values < .05 are highlighted in bold). | | | |
| --- | --- | --- | --- |
| **Networks** | **Mean (Std)** | **Pre-Cortisol** | **Post-Cortisol** |
| **Intra-Hemispheric** | | | |
| TPN/TNN (left) | -1.48 (1.07) | -.261 (.254) | -.175 (.448) |
| TPN/TNN (right) | -1.04 (0.71) | -.263 (.249) | -.080 (.732) |
| TPN/SRN (left) | -0.41 (.81) | -.223 (.331) | -.271 (.235) |
| TPN/SRN (right) | -0.54 (0.77) | -.131 (.571) | -.241 (.293) |
| TNN/SRN (left) | 2.29 (0.90) | .**499 (.021)** | .347 (.123) |
| TNN/SRN (right) | 1.90 (0.91) | .337 (.135) | .025 (.914) |
| **Inter-Hemispheric** |  |  |  |
| TPN (left)/TNN (right) | -1.57 (0.71) | .220 (.337) | -.013 (.955) |
| TPN (left)/SRN (right) | -0.64 (0.70) | -.014 (.952) | -.200 (.385) |
| TPN (right)/TNN (left) | -1.73 (0.66) | -.190 (.408) | -.115 (.621) |
| TPN (right)/SRN (left) | -0.77 (0.86) | -.063 (.788) | -.181 (.433) |
| TNN (left)/SRN (right) | 1.94 (1.07) | **.491 (.024)** | .335 (.137) |
| TNN (right)/SRN (left) | 1.52 (0.81) | **.579 (.006)** | .206 (.369) |

**E. ICC maps and time courses in the supra-threshold voxels in the auditory cortex**

**
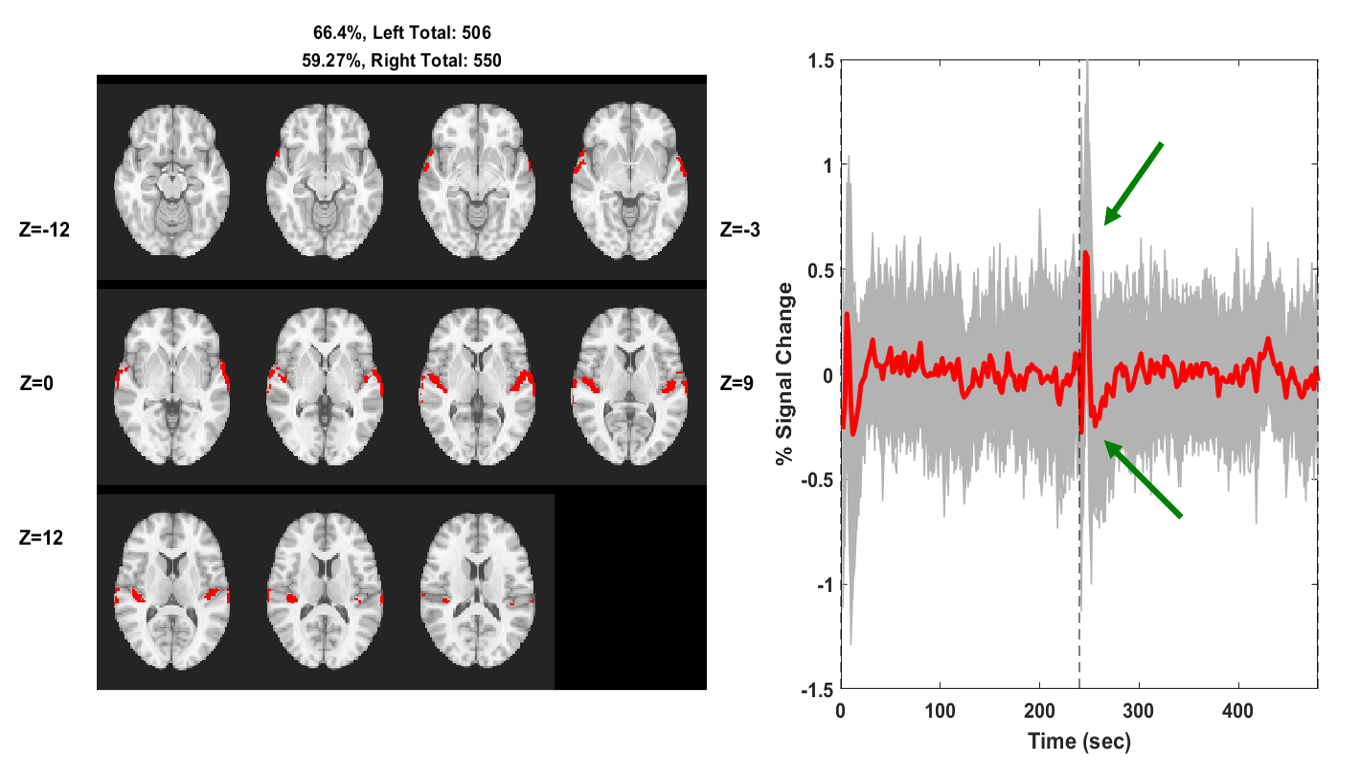
**

Fig. E: Empirical time courses of supra-threshold voxels in the primary auditory cortex (Areas Te1.0, 1.1, 1.2) and Area Te3, which were defined using the JuBrain Atlas. Each time course in grey represents the average of 49 subjects in a supra-threshold voxel. The arrows indicate the increased and decreased activity following the onset of the eyes-open instruction. In the ICC maps, the corresponding supra-threshold voxels are presented in red in a few radiological orientated axial slices with the left hemisphere shown on the right. The total number of voxels in the left and right hemispheres are shown above the maps along with the proportions of supra-threshold voxels.

**F. Values of framewise displacement and derivative of root-mean squared displacement over voxels**


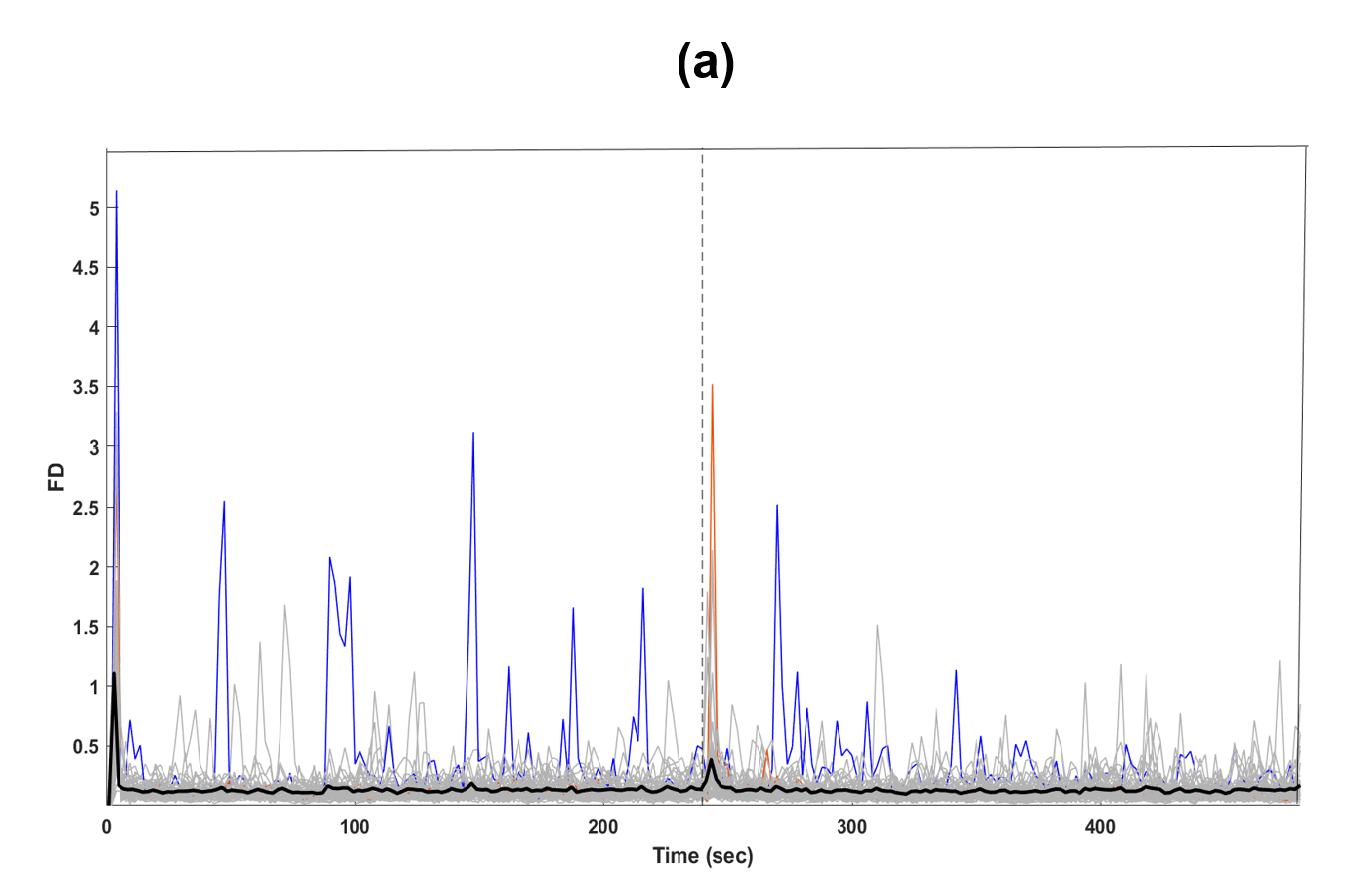


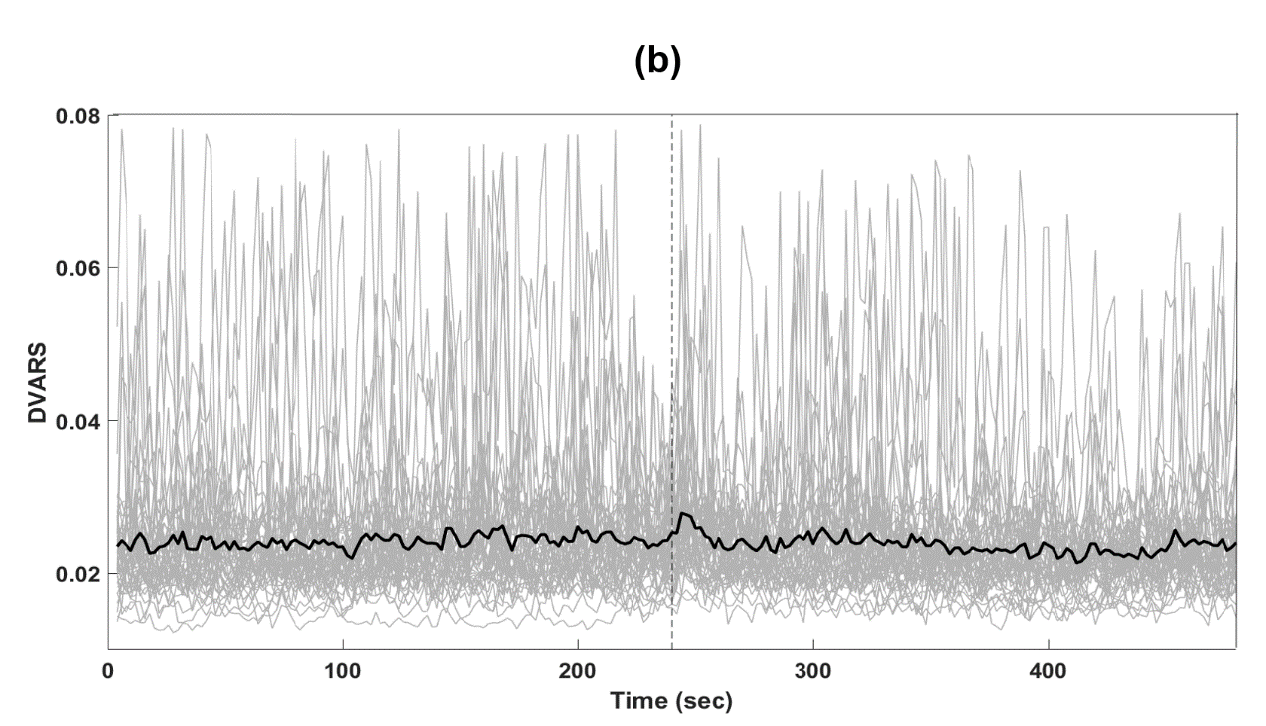


Fig. F: Values of framewise displacement (FD) in (a) and derivative of root-mean squared displacement over voxels (DVARS) in (b) of the 49 subjects receiving EC-EO instructions. The solid time courses are the mean values of the 49 subjects. These highlighted FD values in blue and red are two subjects who had serious head motion after EC/EO onset compared with other subjects.

**G. ICC maps and time courses in the supra-threshold voxels in the hippocampus**

**
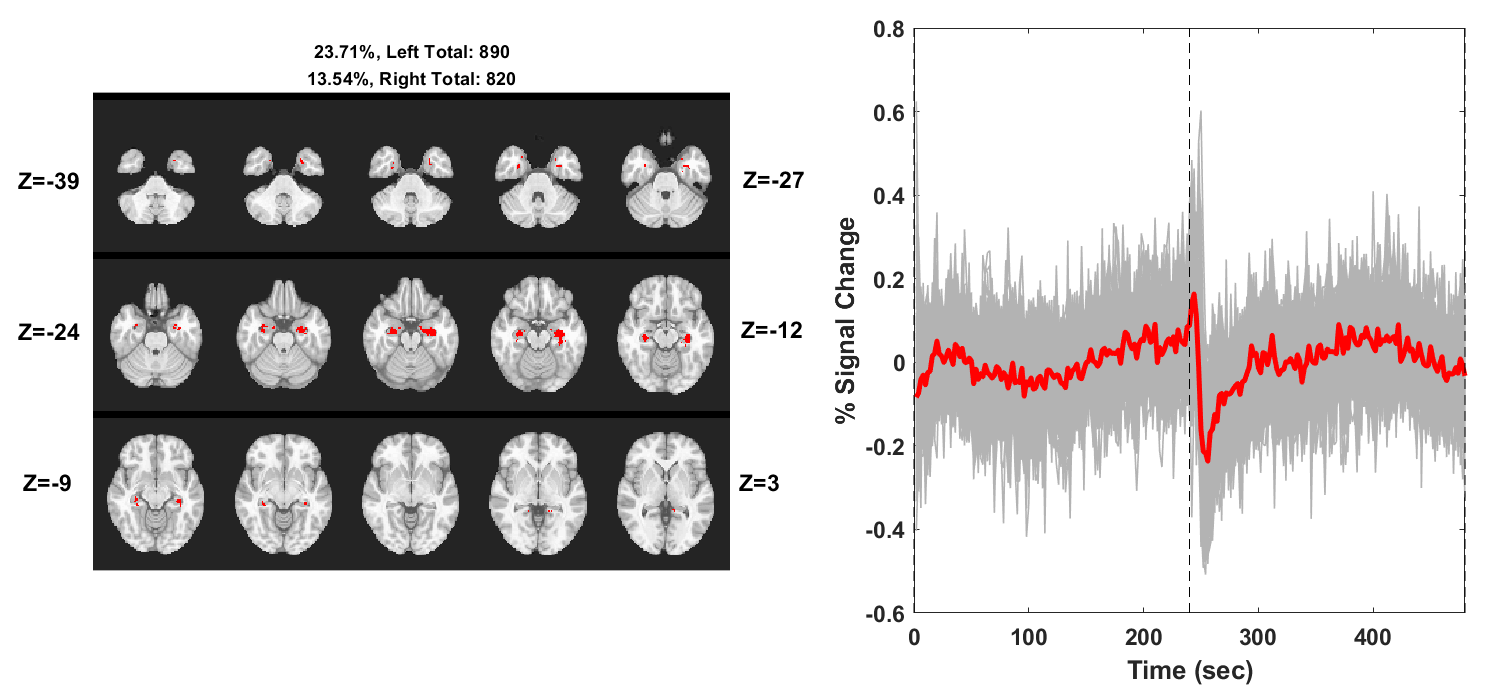
**

Fig. G: Empirical fMRI time courses of supra-threshold voxels in the hippocampus (excluding Area HATA). The areas in the hippocampus were defined using the JuBrain Atlas. Each time course in grey represents the average of 49 subjects in a supra-threshold voxel and the overall average is plotted in red. In the ICC maps, the corresponding supra-threshold voxels in the hippocampus are presented in red in a few radiological orientated axial slices with the left hemisphere shown on the right. The total number of voxels in the left and right hippocampus are shown above the maps along with the proportions of supra-threshold voxels.

**H. Subject-level mean time courses in different networks**

**
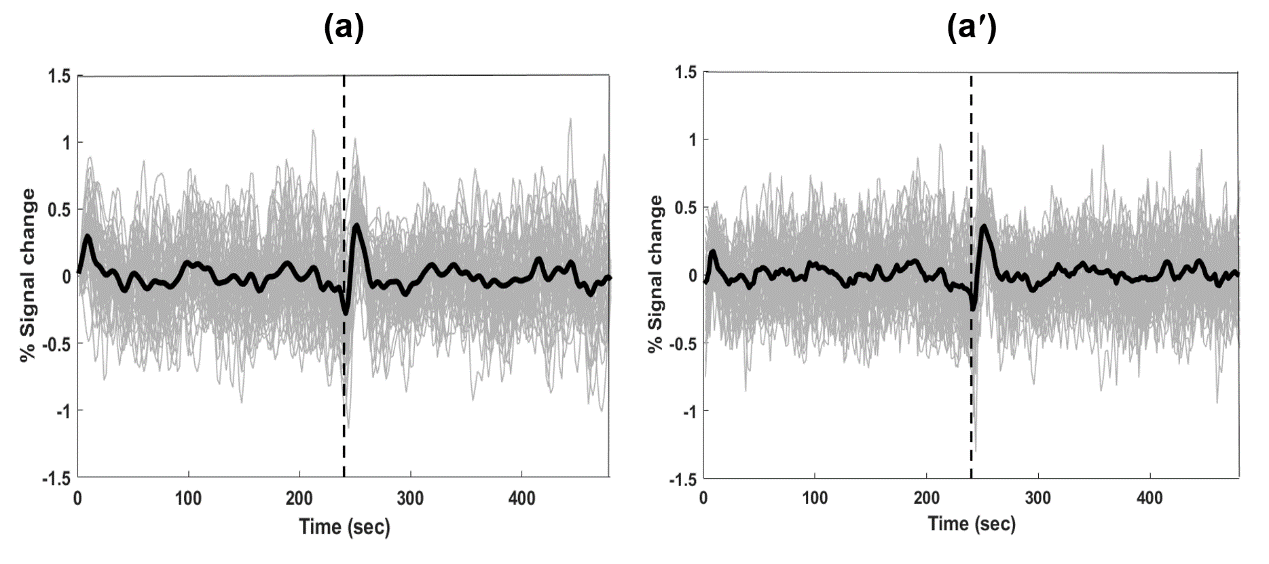

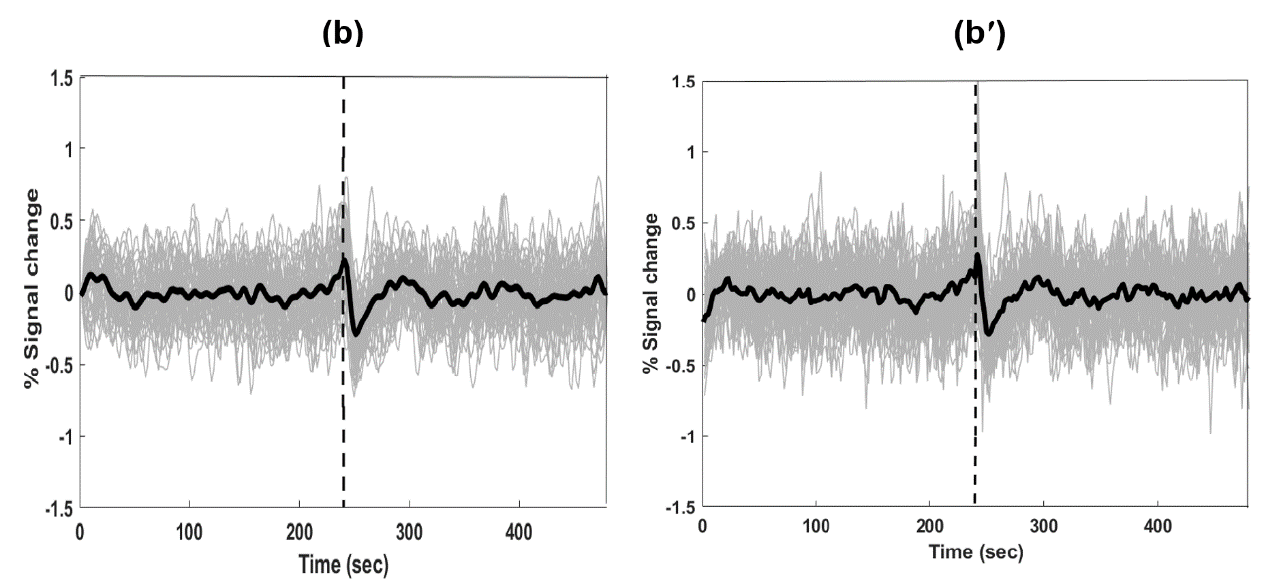

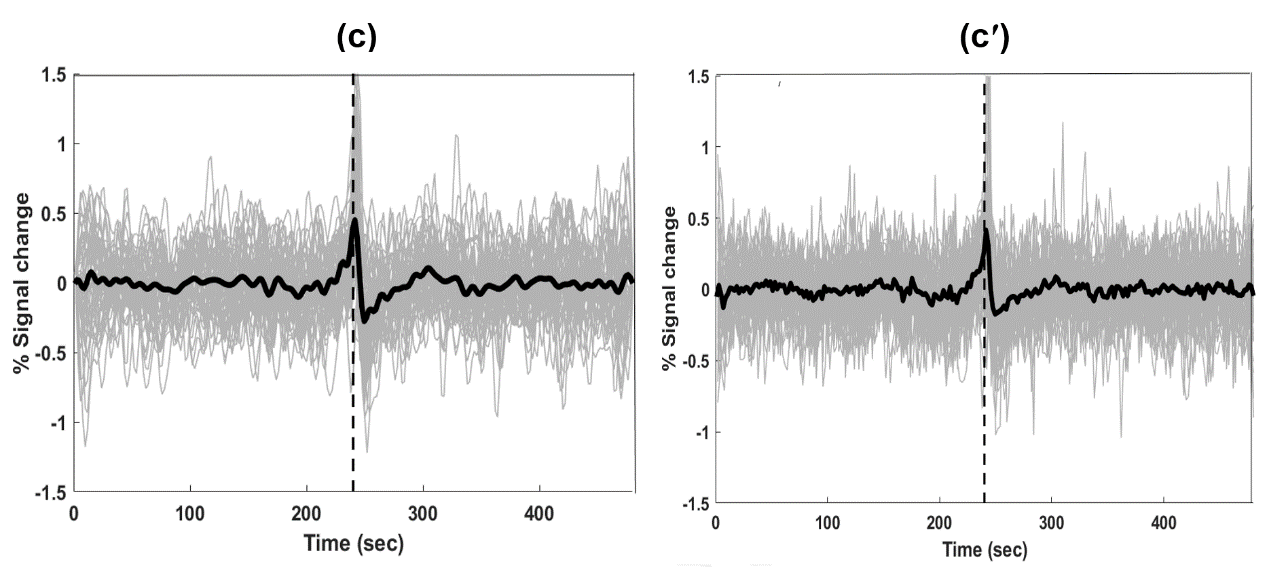
**

Fig. H: Subject-level mean time courses in grey across supra-threshold voxels in the TPN, TNN, and SRN, respectively, in (a), (b) and (c) after bandpass filtering (i.e., 0.01-0.1Hz) and in (aʹ), (bʹ) and (cʹ) after high-pass filtering (i.e., > 0.01Hz). The group-level mean time courses are indicated in bold.
